# A Prospective Cohort Study on the Effects of Repeated Acute Stress on Cortisol Awakening Response and Immune Function in Military Medical Students

**DOI:** 10.3390/biomedicines12112519

**Published:** 2024-11-04

**Authors:** Madison A. Propp, Dean Paz, Sukhrob Makhkamov, Mark E. Payton, Qamrul Choudhury, Melodie Nutter, Rebecca Ryznar

**Affiliations:** 1 College of Osteopathic Medicine, Rocky Vista University, 8401 S Chambers Rd, Englewood, CO 80112, USA; 2 Department of Emergency Medicine, University of Texas at Austin Dell, 1500 Red River St, Austin, TX 78701, USA; 3 Department of Biomedical Sciences, College of Osteopathic Medicine, Rocky Vista University, 8401 S Chambers Rd, Englewood, CO 80112, USA; mpayton@rvu.edu (M.E.P.); qchoudhury@rvu.edu (Q.C.); rryznar@rvu.edu (R.R.); 4 Arizona College of Nursing, 8363 West Sunset Road, Las Vegas, NV 89113, USA; menutter@arizonacollege.edu

**Keywords:** cortisol awakening response (CAR), repeated acute stress, salivary cytokines, immune function, hyper-realistic surgical simulation, stress physiology

## Abstract

**Background:** The cortisol awakening response (CAR) is a pivotal component of the body’s stress response, yet its dynamics under repeated acute stress and its interplay with immune biomarkers remain inadequately understood. **Methods:** This study examined 80 second-year military medical students undergoing a 5-day intensive surgical simulation designed to elicit stress responses. Salivary samples were collected daily upon waking and 30 min thereafter to measure cortisol and a panel of cytokines using bead-based multiplex ELISA. **Results:** Analysis revealed a significant blunting of the CAR on the third day of training (*p* = 0.00006), followed by a recovery on the fourth day (*p* = 0.0005). Concurrently, specific cytokines such as CXCL1 (r = 0.2, *p* = 0.0005), IL-6 (r = 0.13, *p* = 0.02), IL-10 (r = 0.14, *p* = 0.02), and VEGF-A (r = 0.17, *p* = 0.003) displayed patterns correlating with the CAR, with increased strength of associations observed when assessing cytokine levels against the CAR of the preceding day (CXCL1 r = 0.41, *p* = 0.0002. IL-6 r = 0.38, *p* = 0.0006. IL-10 r = 0.3, *p* = 0.008. VEGF-A r = 0.41, *p* = 0.0002). **Conclusions:** These results suggest a temporal relationship between stress-induced cortisol dynamics and immune regulation. The CAR pattern demonstrated in this study may represent induction of and recovery from psychological burnout. Moreover, the observed cytokine associations provide insight into the mechanisms by which stress can influence immune function. The results may have broader implications for managing stress in high-performance environments, such as military and medical professions, and for identifying individuals at risk of stress-related immune suppression.

## 1. Introduction

Stress is an unavoidable part of our lives but the impacts of repeated stressful events on our immune system and physiology are not completely understood. Understanding the interplay between stress and immune system activation or suppression is crucial, as imbalances can lead to various pathologies. This research contributes to better prevention, recognition, and treatment of psychological, infectious, autoimmune, and other disorders.

The stress response is modulated by two systems: the hypothalamic–pituitary–adrenal (HPA) axis and the autonomic nervous system (ANS). These two systems help to address stress and return an organism to a homeostatic baseline [1]. Disruptions to these systems underlie many pathologies including anxiety, depression, autoimmune conditions, and more [1]. The ANS is primarily concerned with managing acute changes to the environment such as threats or rewards and managing the balance between “fight or flight” responses and “rest and digest” responses [1]. Meanwhile, the HPA axis is concerned with management of the stress response and regular cycles the organism undergoes, such as cycles related to growth, energy management, and reproduction [1]. Both systems modulate activities all over the body, often through chemical messengers, such as hormones and cytokines, that are released into bodily fluids such as blood and saliva [1]. Cytokines have a diverse variety of functions, including various components of the immune response, wound healing, angiogenesis, and energy mobilization. Concentrations of these biomarkers may be measured to quantify the activity of these systems [2]. Saliva is the most convenient and noninvasive bodily fluid to obtain from participants, and salivary cytokine concentrations have been shown to reflect serum concentrations [2,3]. 

Cortisol, the principal glucocorticoid in humans, is a steroid hormone released both in daily cycles, including the cortisol awakening response (CAR) [4], and acutely in response to stress as a part of the HPA axis [5]. While cortisol is prototypically considered anti-inflammatory, as evidenced by using exogenous glucocorticoids to treat conditions like COPD and chronic pain disorders, its impact on the immune system is complex and varied [6]. Patients on long-term steroid therapy are often immunocompromised, necessitating precautions against infectious diseases.

One aspect of the stress immune relationship that warrants further investigation is the cortisol awakening response. The cortisol awakening response (CAR) is a distinct rise of approximately 50–80% in cortisol levels within the first 30–40 min of waking [7]. This response has demonstrated consistent intra-personal reliability from day to day [8,9] and is hypothesized to prepare the organism for the anticipated stresses of the coming day [1,10,11]. Alterations in CAR have been associated with various pathologies, including autoimmune disease, cardiovascular disease, chronic pain disorders, multiple sclerosis, PTSD, and depression [11,12,13]. CAR has also been shown to be impacted by psychological stressors in humans [14,15] and physical stressors in rodents [11]. However, little is known about the relationship between the CAR itself and immune function. 

More broadly, stress is a known modifier of the immune system. Stressors and their corresponding immune effects have been classified by temporality; acute time-limited stressors such as public speaking typically upregulate innate immunity [16], prioritizing a swift response, while brief naturalistic stressors such as academic exams cause alterations to cytokine profiles consistent with shifting from cellular immune responses to humoral immune responses, suggesting the body is primed to address extracellular pathogens [16]. Chronic stressors such as unemployment or disability led to global immunosuppression [16], leaving individuals vulnerable to infection. Cortisol itself appears to sensitize the body to respond to acute threats; higher levels of cytokine expression are seen after lipopolysaccharide (LPS) administration when cortisol levels are high compared to when cortisol levels are low [17]. Additionally, glucocorticoids have been shown to mediate leukocyte shifts, mobilizing neutrophils to the blood in anticipation of injury. Animal models suggest that this system is protective against autoimmunity with concomitant impaired viral immunity [18]. 

Due to the scarcity of controlled environments for stress studies on human subjects, most research on stress responses predominantly relies on animal models that induce stress via restraint or injury. Our study utilized a rather unique protocol. Annually, military medical students engage in an intensive training experience at Strategic Operations^TM^ (STOPS) in San Diego, CA, USA, called Intensive Surgical Skills Week (ISSW). ISSW is a series of hyper-realistic^®^ mass casualty and operating room simulations created to train students for battlefield and emergency scenarios, utilizing special effects, human-worn surgical simulators (Cut Suits), and high-fidelity mannequins over the course of 5 days. This training is designed to be immersive, induce stress responses, and inoculate students to the adverse effects of stress on cognition and performance [19]. Previous cohorts have demonstrated decreased perceived stress, increased confidence, and improvements in multiple skills over the course of the week [20]. Additionally, this course has been shown to influence salivary levels of stress-related cytokines [21]. Students go through each simulation in a different order depending on their assigned group, but all students go through the same scenarios twice with no changes to the scenario protocols between years [22]. Students stay in barracks on site and follow consistent lights out and wake times during the training. 

Most human subject studies on CAR have been conducted in noncontrolled environments or over short periods, typically two consecutive days, and often with small sample sizes. In this study, we aim to evaluate CAR across four days in a relatively large sample under controlled conditions previously shown to cause repeated acute stress in healthy individuals [19]. Additionally, we seek to explore the relationships between CAR, repeated acute stress, and immune cytokines. We theorize that, initially, CAR will increase to address the acute daily stress and then attenuate over the course of the week as students are habituated to the stressful environment. We anticipate negative correlations between CAR and classically inflammatory cytokines such as IL-6, TNFα, and IL-1β. 

This research enhances our understanding of human stress responses and their impact on immune function. This study will help us to identify broad patterns of cytokine signaling in stressful environments. This study may also contribute to the identification of individuals more susceptible to immune suppression due to stress and ways to monitor them more appropriately or take steps to prevent infection in these individuals. This research may also contribute to our knowledge of how stress impacts autoimmune conditions and to the understanding and treatment of mental health, cardiovascular, and other stress-related pathologies.

## 2. Materials and Methods

All protocols were reviewed and approved by the Rocky Vista University Institutional Review Board, protocol number #2019-108. Eighty 2nd-year military medical students (63.75% male, mean age = 26.6, range 23–32) from Rocky Vista University College of Osteopathic Medicine, Touro College of Osteopathic Medicine, Noorda College of Osteopathic Medicine, and Western University of Health Sciences without known health problems provided informed consent. Participants (Table 1) engaged in Intensive Surgical Skills Week (ISSW), a hyper-realistic mass casualty and surgical simulation course designed to induce stress and train participants for emergency/battlefield medicine over 5 days. Data were collected during two iterations of ISSW in May 2022 and May 2023 at STOPS in San Diego, CA, with no alterations to the content of training between years. Cohorts were combined to assess dynamics across all cohorts.

**Figure 1:**
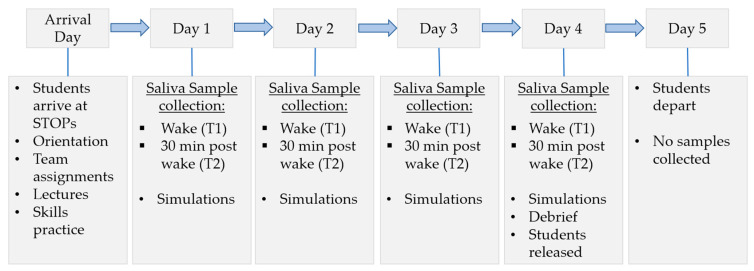
Intensive Surgical Skills Week schedule. Samples were collected at wake and 30 min post waking. Students go through hyper-realistic mass casualty and operating room simulations, going through each simulation two times over the duration of training.

The first day of training entailed arrival to the site, orientation, and lectures. Sample collection began on the second morning of training (hereafter referred to as Day 1), prior to any stressful scenarios, and occurred daily until the completion of training (Day 4). As the first sample was collected before the onset of the repeated acute stressors, participants can serve as their own pre-stress controls. See Figure 1 for schedule details. Participants gave an initial saliva sample upon waking (Time 1) and a second sample 30 min later (Time 2). Participants were instructed not to eat, drink, or brush their teeth until after the daily sampling was complete. Saliva was collected using the whole stimulated saliva method as described in Ryznar et al. [23]. Briefly, participants rinsed their mouth with water, chewed sugar free gum, then provided 1 mL of saliva which was pipetted into a 1.5 mL microcentrifuge tube labeled with the participant ID and sample time. Grossly bloody or mucoid samples were discarded. A 1 μg/mL concentration of protease inhibitor was added to the samples which were then placed on ice (20–201 Millipore, Burlington, MA, USA) for storage and subsequently shipped on dry ice to Eve Technologies (Calgary, AB, Canada) for analysis.

Bead-based multiplex ELISA panels of cytokines/chemokines and cortisol were used to quantify biomarker concentration in each sample. In the 2022 cohort (n = 40), a custom 16-plex human cytokine panel (EGF, FGF-2, CXCL1, IL-1α, IL-1RA, IL-6, IL-8, IL-10, IL-13, IL-15, IL-18, IL-27, MCP-1, PDGF-AA, TGF, and VEGF-A) and multi-species 1-plex custom hormone panel (cortisol) were completed (Millipore Sigma, Burlington, MA, USA). For the 2023 samples (n = 40), a 21-plex custom human cytokine panel (all of the above cytokines plus TNFα, CX3CL1, G-CSF, INF-γ, and IL-1β) and 2-plex human circadian stress panel (cortisol and melatonin) were completed (Millipore Sigma, Burlington, MA, USA). The multiplexing analysis was performed using the Luminex™ 200 system (Luminex, Austin, TX, USA) by Eve Technologies Corp. (Calgary, AB, Canada). Assay sensitivities of these markers range from 4.3 to 686 pg/mL for the 1- and 2-plex panels. Individual analyte sensitivity values are available in the Millipore Sigma MILLIPLEX^®^ MAP protocol HNCSMAG-35K. Assay sensitivities for the 21-plex and 16-plex panels range from 0.14 to 50.78 pg/mL. Individual analyte sensitivity values are available in the Millipore Sigma MILLIPLEX^®^ protocol HCYTA-60K.

### Statistical Analysis

Data from the two cohorts were pooled and grouped by participant ID, collection day, and time. Extrapolated and low bead count values were removed, while out-of-range values were replaced with the upper limit of the reference range or zero for values exceeding or falling below the range, respectively. IL-13, IL-27, and melatonin were excluded from further analysis due to many extrapolated and out-of-range values (312 of 640 samples for IL-13, 422 of 640 samples for IL-27, and 230 of 320 samples for melatonin). One participant was missing data for multiple sample time points and was excluded from analysis, bringing the total sample size to seventy-nine participants. If values were missing for a given calculation, they were excluded from that calculation.

All statistical analyses were performed using SAS Version 9.4 [24] or GraphPad Prism for Windows Version 10.2.3, and all figures were generated using GraphPad Prism for Windows version 10.2.3 and Microsoft® PowerPoint® for Microsoft 365 MSO (Version 2410) [25,26]. Power analysis was performed, and given our sample size, we can find a difference in means that is one-half of a standard deviation with 0.72 probability. Cortisol awakening response (CAR) was calculated as the percent change in salivary cortisol between Time 1 (waking) and Time 2 (30 min post waking). One-way repeated-measures analysis of variance (ANOVA) and subsequent paired *t*-tests were used to compare the mean CAR of all participants across the 4 days of sampling. Repeated-measures ANOVAs and subsequent Tukey’s multiple-comparison tests were run comparing average values for each cytokine at each time point. Pearson correlations were calculated between individual CARs and average of Time 1 and Time 2 cytokine values, as well as Time 1 and Time 2 cytokine values separately. Given the mechanism of action of steroid hormones is typically delayed due to the time it takes to regulate gene transcription [5], Pearson correlations between CAR on a given day and cytokine levels on the following day were also performed. Cytokine descriptive statistics may be found in the Supplementary Materials.

## 3. Results

**Figure 2:**
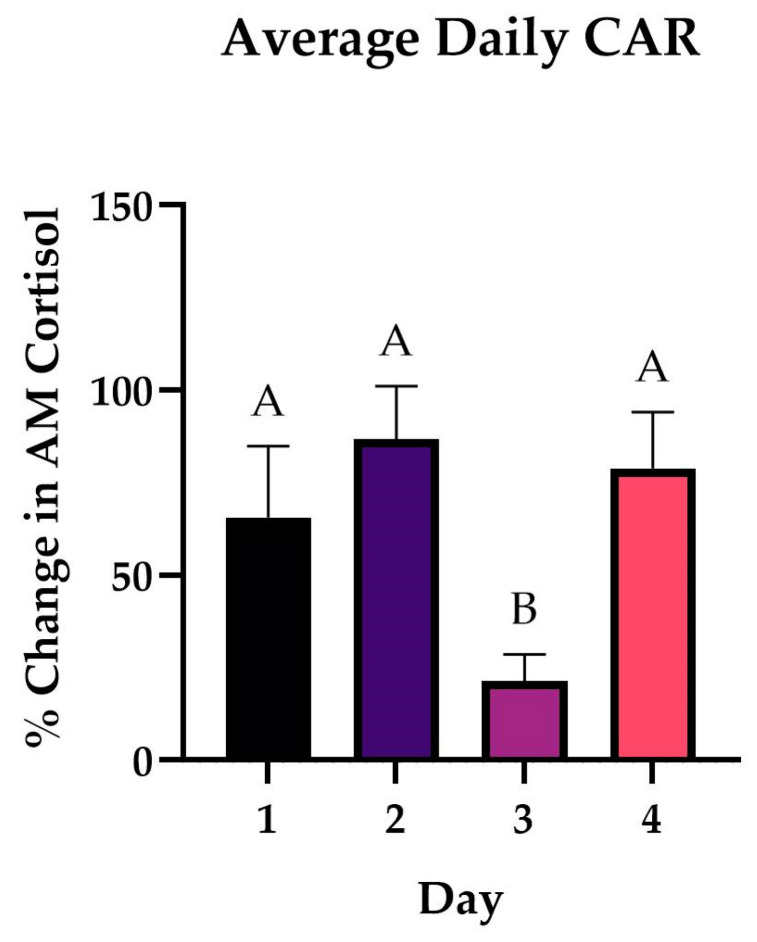
Daily mean percent change in AM salivary cortisol from waking to 30 min later (CAR) across all participants (n = 79) during repeated acute stress. Error bars represent the standard error of the mean (SEM). Results of pairwise comparisons between daily means are shown by the compact letter display. Two means with the same letter are not significantly different at the 0.05 level. Day 3 CAR is significantly reduced compared to all other days (D1, D3 *p* = 0.014), (D2, D3 *p* = 0.00006), (D4, D3 *p* = 0.0005). Day 1 average CAR = 67.28%, Day 2 = 84.45%, Day 3 = 21.30%, Day 4 = 78.83%. Typically, a healthy CAR is expected to be between 50 and 80%.

ANOVA revealed significant differences in mean cortisol response between days (*p* = 0.01). Subsequent *t*-tests between CAR Day 1/CAR Day 3 (*p* = 0.014), CAR Day 2/CAR Day 3 (*p* = 0.00006), and CAR Day 3/CAR Day 4 (*p* = 0.0005) showed significant differences. It is noteworthy that an ANOVA comparing Time 1 cortisol across all 4 days did not show significant differences between Day 1 and Day 3 (*p* = 0.59), Day 2 and Day 3 (*p* = 0.13), or Day 3 and Day 4 (*p* = 0.86), indicating the observed CAR differences were not contributable to changes in baseline values and rather reflect genuine differences in this specific aspect of the HPA axis. The average CARs across all participants each day with SEM and multiple comparisons were plotted (Figure 2). 

**Figure 3:**
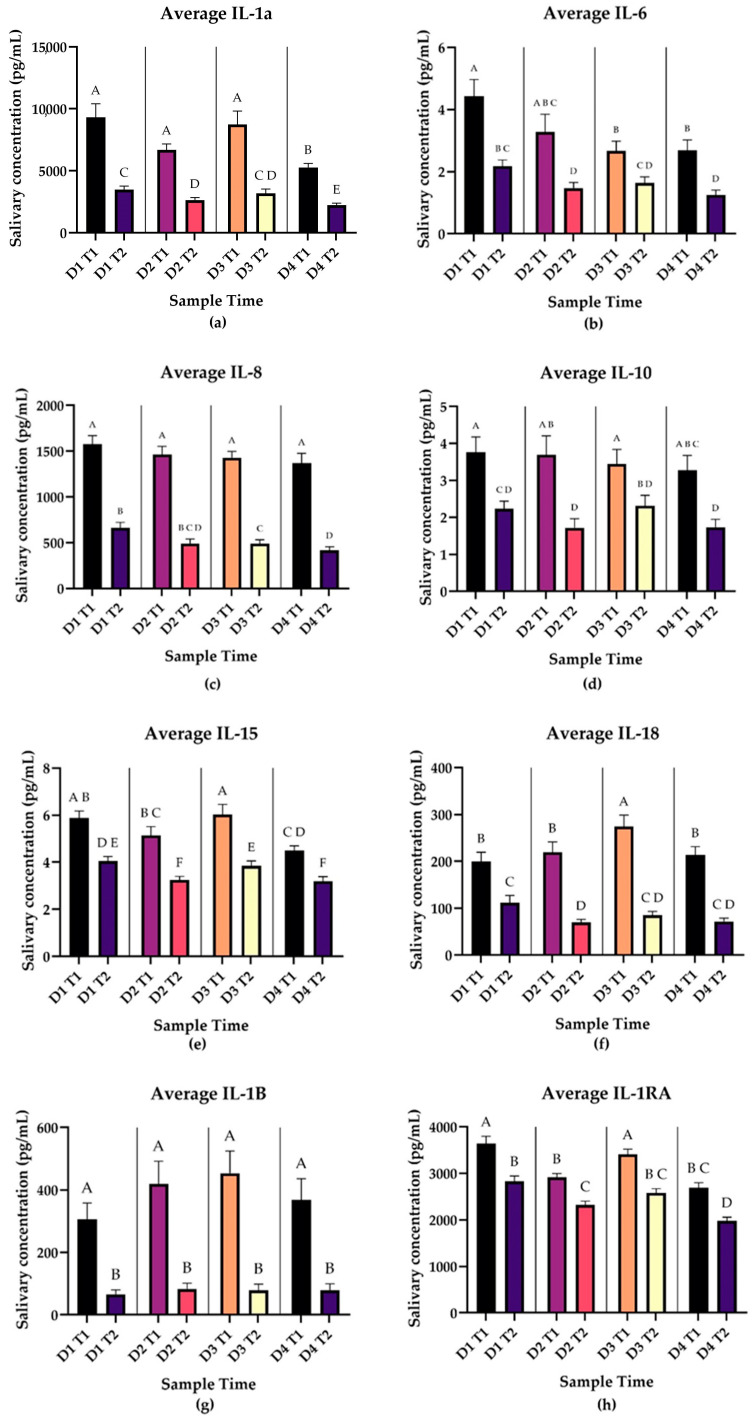
Panels A-H represent daily average salivary interleukin levels in pg/mL at Time 1 (waking) and Time 2 (30 min post waking) during repeated acute stress. Error bars represent the SEM. Results of pairwise comparisons between average cytokine values at each time point are shown by the compact letter display. Two means with the same letter are not significantly different at the 0.05 level. All interleukins exhibited significant decreases in average salivary concentration from Time 1 to Time 2 in all days of the sampling period. IL-1a, IL-8 Il-15, IL-18, and IL-1RA, *p* < 0.0001. IL-6 Day 1, *p* = 0.0014; Day 2, *p* = 0.0124; Day 3, *p* = 0.0003; Day 4, *p* < 0.0001. IL-10 Day 1, *p* = 0.003; Day 2, *p* = 0.0056; Day 3, *p* = 0.0086; Day 4, *p* = 0.0098. IL-1B Day 1, *p* = 0.0001; Days 2–4, *p* < 0.0001. (**a**) IL-1a (n = 79), (**b**) IL-6 (n = 79), (**c**) IL-8 (n = 79), (**d**) IL-10 (n = 79), (**e**) IL-15 (n = 79), (**f**) IL-18 (n = 79), (**g**) IL-1β (n = 40), (**h**) IL-1RA (n = 79).

Average daily values for each cytokine at Time 1 and Time 2 were calculated along with ANOVAs and Tukey’s tests comparing average cytokine levels at each time point. Averages and comparisons were plotted with SEM for interleukins and interleukin-associated cytokines (Figure 3). All exhibited significant decreases in average salivary concentration from Time 1 to Time 2 each day. IL-1a, IL-8 Il-15, IL-18, and IL-1RA had a *p* < 0.0001. For IL-6, on Day 1, *p* = 0.0014; Day 2, *p* = 0.0124; Day 3, *p* = 0.0003; and Day 4, *p* < 0.0001. For IL-10, on Day 1, *p* = 0.003; Day 2, *p* = 0.0056; Day 3, *p* = 0.0086; and Day 4, *p* = 0.0098. For IL-1B, on Day 1, *p* = 0.0001, while for Days 2–4, *p* < 0.0001. Complete results of multiple-comparison tests may be found in the Supplementary Materials. 

This decrease in the post wake period is the opposite of the classical CAR. This suggests that these cytokines are under circadian patterning in their release as well.

**Figure 4:**
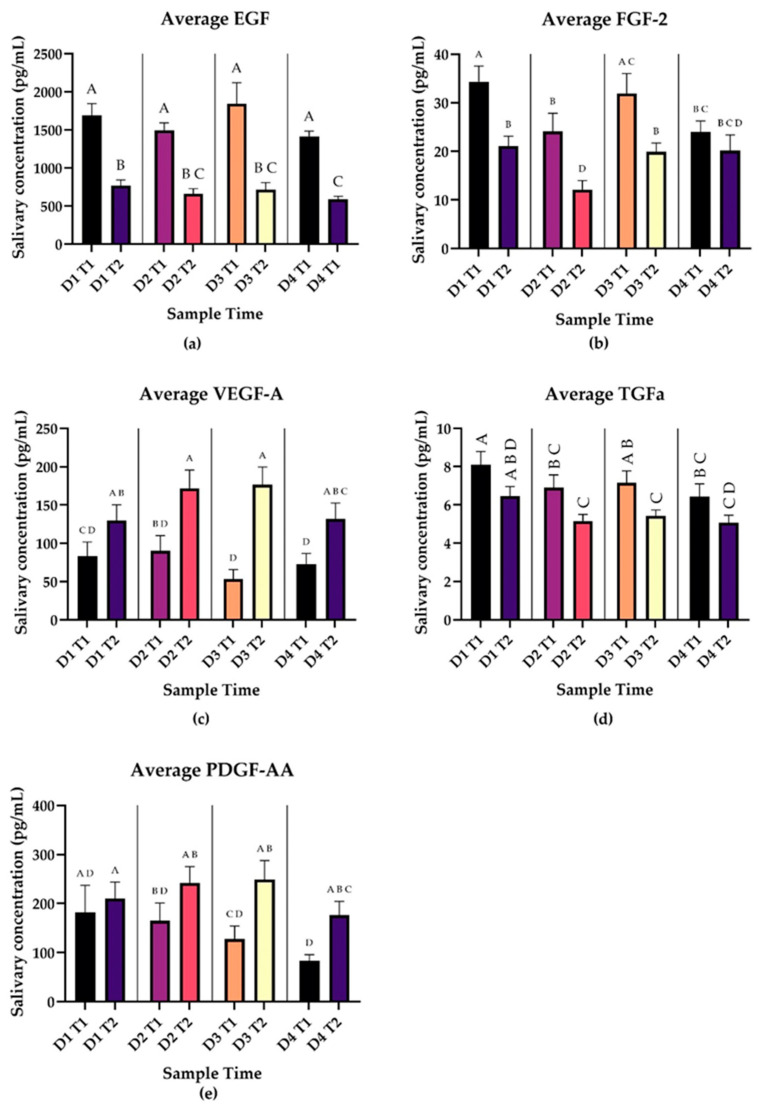
Panels A-E represent daily average salivary growth factor levels in pg/mL at Time 1 (waking) and Time 2 (30 min post waking) during repeated acute stress. Error bars represent SEM. Results of pairwise comparisons between average cytokine values at each time point are shown by the compact letter display. Two means with the same letter are not significantly different at the 0.05 level. VEGF-A showed significant increases in the post wake period on all 4 days (*p* = 0.0059, *p* = 0.0105, *p* < 0.0001, *p* = 0.0155). PDGF-AA showed significant increases in the post wake period on Days 3 and 4 (*p* = 0.0001, *p* = 0.0005). EGF, showed significant decreases in the post wake period on all days (*p* < 0.0001). FGF-2 showed significant decreases on Days 1–3 (*p* < 0.0001, *p* = 0.0003, *p* = 0.008). TGFα showed a significant decrease on Day 3 (*p* = 0.011). (**a**) EGF (n = 79), (**b**) FGF-2 (n = 79), (**c**) VEGF-A (n = 79), (**d**) TGFα (n = 79), (**e**) PDGF-AA (n = 79).

Growth factors (Figure 4) showed variable patterns from Time 1 to Time 2. VEGF-A showed significant increases in the post wake period on all 4 days (*p* = 0.0059, *p* = 0.0105, *p* < 0.0001, *p* = 0.0155). PDGF-AA showed significant increases in the post wake period on Days 3 and 4 (*p* = 0.0001, *p* = 0.0005). In contrast, EGF showed significant decreases in the post wake period on all days (*p* < 0.0001). FGF-2 showed significant decreases on Days 1–3 (*p* < 0.0001, *p* = 0.0003, *p* = 0.008). TGFα showed a significant decrease on Day 3 (*p* = 0.011). Complete results of multiple comparisons may be found in the Supplementary Materials.

VEGF-A and PDGF-AA move in the same direction as cortisol in the post wake period while EGF, FGF-2, and TGFα move in the opposite direction as cortisol in the post wake period.

**Figure 5:**
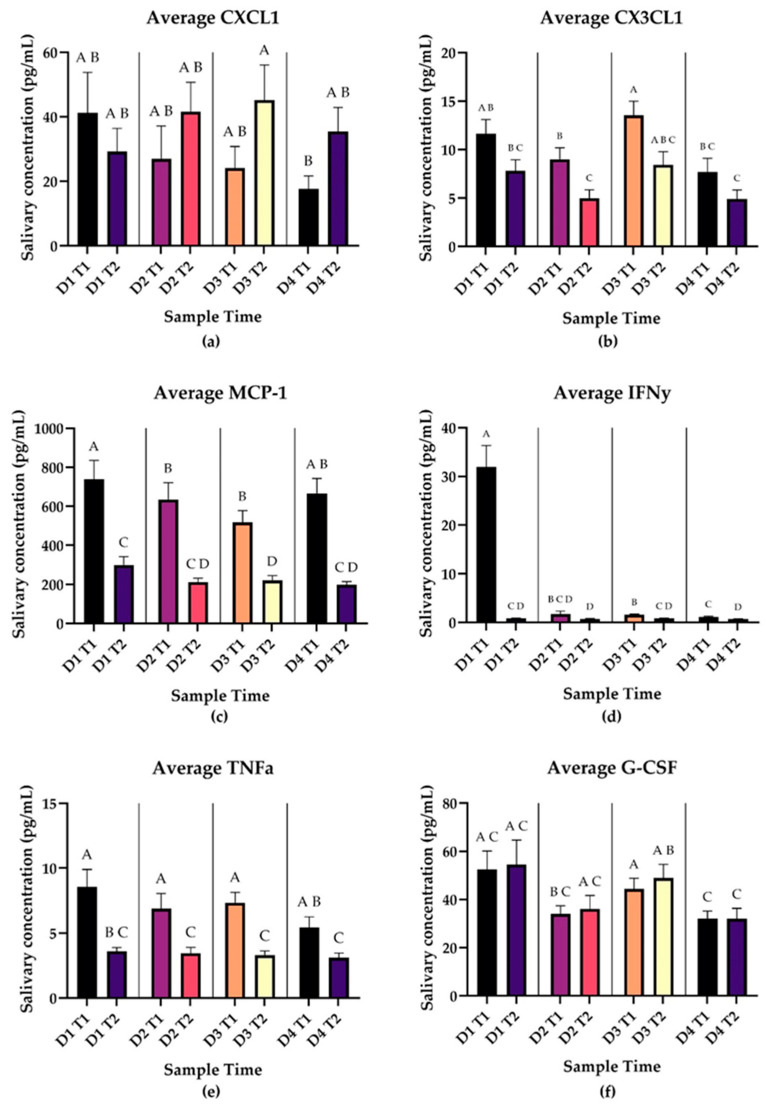
Panels A-F represent daily average salivary chemokine and miscellaneous cytokine levels in pg/mL at Time 1 (waking) and Time 2 (30 min post waking) during repeated acute stress. Error bars represent SEM. Results of pairwise comparisons between average cytokine values at each time point are shown by the compact letter display. Two means with the same letter are not significantly different at the 0.05 level. MCP-1 showed significant decreases in the post wake period on all 4 days (*p* < 0.0001). CX3CL1 showed decreases in salivary concentration in the post wake period on all days but only the decrease on Day 2 reached statistical significance (*p* = 0.0198). TNFα showed significant decreases in salivary concentration from Time 1 to Time 2 on all days (*p* = 0.0021, *p* = 0.0055, *p* < 0.0001, *p* = 0.0028). G-CSF levels do not show significant changes from Time 1 to Time 2 on any day; however, overall levels are significantly decreased between Day 3 and Day 4 (T1 *p* = 0.0028, T2 *p* = 0.0001). (**a**) CXCL1 (n = 79), (**b**) CX1CL3 (n = 40), (**c**) MCP-1 (n = 79), (**d**) IFNγ (n = 40), (**e**) TNFα (n = 40), (**f**) G-CSF (n = 40).

CXCL1 levels decrease between T1 and T2 on Day 1 and then increase between T1 and T2 on Days 2–4, but none of the changes between Time 1 and Time 2 were statistically significant (Figure 5). MCP-1 showed significant decreases in the post wake period on all 4 days (*p* < 0.0001). CX3CL1 showed decreases in salivary concentration in the post wake period on all days but only the decrease on Day 2 reached statistical significance (*p* = 0.0198). TNFα showed significant decreases in salivary concentration from Time 1 to Time 2 on all days (*p* = 0.0021, *p* = 0.0055, *p* < 0.0001, *p* = 0.0028). G-CSF levels do not show significant changes from Time 1 to Time 2 on any day; however, overall levels are significantly decreased between Day 3 and Day 4 (T1 *p* = 0.0028, T2 *p* = 0.0001). Complete multiple-comparisons data may be found in the Supplementary Materials. 

Salivary IFNγ concentrations show a significant decrease between Day 1 Time 1 and Day 1 Time 2; salivary levels remain at this lower level for the remainder of sampling.

Pearson correlation coefficients were calculated to evaluate the relationship between CAR and levels of each biomarker. Several significant but weak associations between CAR and CXCL1, IL-6, IL-10, and VEGF-A were found (Table 2). When comparing cytokine levels to the previous day’s CAR, Pearson correlations revealed multiple significant moderate relationships (Table 3 and Table 4). When average CAR was normal to elevated on Day 2, we see stronger relationships with CXCL1, IL-6, IL-10, and VEGF-A at Day 3 Time 1. When comparing the blunted CAR on Day 3 to Day 4 Time 1 cytokines, we observed a positive correlation with EGF, and a negative correlation with FGF-2.

## 4. Discussion

Our study adds further evidence to the multifaced role of the CAR in the management of stress and its interactions with the immune system. We identified a unique pattern of CAR in our subjects undergoing repeated acute stress with significant blunting of the response on Day 3 and return-to-normal range on Day 4. Most cytokines showed decreases in concentration from Time 1 to Time 2 (Figure 3, Figure 4 and Figure 5). VEGF-A and PDGF-AA showed consistent increases from T1 to T2 for the duration of sampling (Figure 3), while CXCL1 and G-CSF showed increases from T1 to T2 intermittently throughout the week (Figure 5), which is in association with the pattern of secretion of morning cortisol. This suggests that a properly functioning CAR may be a component of wound healing and growth factor expression. 

Cytokines including CXCL1 and IFNγ showed alterations in patterning later in the week, similar to changes to the CAR. CXCL1 shows a significant decrease from Day 3 Time 2 to Day 4 Time 1 (Figure 5a), which may be related to the blunting of the CAR on Day 3. It is noteworthy that IFNγ shows a very different pattern compared to the rest of the cytokines, peaking during the initial sampling, drastically decreasing at Time 2, and remaining low for the remainder of the sampling period (Figure 5d). This is consistent with previously documented findings regarding IFNγ’s relationship to psychological stress; IFNγ secretion has been shown to be inhibited by glucocorticoid release in the context of restraint stress in mice [27]. IFNγ is a classical anti-viral cytokine, though it plays an important role in bacterial and parasite immunity as well [28]. This drastic lowering of IFNγ levels during repeated acute stress has large implications for vaccine-based immunity and for medical professionals continuously exposed to pathogens, which merits further investigation.

We also demonstrated correlations between the CAR and the cytokines CXCL1, IL-6, IL-10, and VEGF-A (Table 2). These associations were strengthened by analysis of the CAR as a lag variable with a 24 h delay comparing Day 2 CAR to Day 3 Time 1 cytokines (Table 3). The lag analysis also revealed a positive association with Day 3 CAR and Day 4 Time 1 EGF, and a negative association with Day 4 Time 1 FGF-2 (Table 4).

Interestingly, almost all cytokines showed changes in salivary level in the post wake period, some with remarkable consistency (see Figure 3, Figure 4 and Figure 5). Our findings suggest that components of the immune system also undergo circadian rhythms, demonstrating other “awakening responses”, and represent a valuable area for further investigation. Previous studies have identified that nearly all immune cells undergo circadian cycles, regulated both locally by self-regulating clock genes and centrally by the suprachiasmatic nucleus (SCN) [29]. This daily cycle is further seen in consistent time-of-day symptomatology patterns in asthma, allergies, and rheumatoid arthritis [29]. These clock genes show promise as targets for several therapies, and awareness of these daily cycles is proving beneficial regarding timing of vaccination and medication administration [30]. 

Our study aligns with the existing literature regarding stress and the CAR. Other studies have found a decreased CAR in the context of burnout [31] as well as in police officers experiencing significant occupational stressors [32]. Chronic stress has also been shown to induce glucocorticoid receptor resistance [33]. However, to our knowledge, this is the first study to observe a blunting and recovery of the CAR in the context of repeated acute stress.

The majority of cytokines associated with the CAR in our study (CXCL1, IL-6, IL-10, VEGF-A, and FGF-2) have established relationships to stress in the literature. CXCL1, also known as Gro-α, is an important chemokine that recruits neutrophils from the bone marrow to the blood [34], preparing the organism for possible breaches of the skin (e.g., a cut). In mice experiencing acute restraint stress, CXCL1 levels were found to be under the influence of the HPA axis and directly associated with blood neutrophilia [18]. 

IL-6 is a pleotropic cytokine typically released in the context of infection and tissue damage induced by toll-like receptors (TLRs) and damage-associated molecular proteins (DAMPs), often in association with IL-1β and TNFα [35]. IL-6 has metabolic effects on the liver which promotes gluconeogenesis but dampens the inflammatory response and peaks in the blood 16 h later than cortisol after a stressful event [36]. Other studies have found an association between increased IL-6, perceived stress, and flattened diurnal cortisol slopes [37]. Additionally, sleep-restricted firefighters with higher morning IL-6 levels had higher evening cortisol. Furthermore, all firefighters, regardless of sleep restrictions, with high morning IL-6 had higher daily cortisol levels [38]. In contrast to our findings, another study identified that higher IL-6 was associated with a less pronounced CAR; however, these results are from older participants on typical weekdays not undergoing acute repeated stress [39]. 

IL-10 is classically considered the principle anti-inflammatory cytokine, though it appears to have a more nuanced role in the inflammatory response than previously indicated [40]. It has been shown to be a key player in wound healing. In another study, participants with higher IL-10 levels were found to have flatter cortisol decline slopes [39]. Also, circadian misalignment increased IL-10, TNFα, and CRP levels [41]. This is consistent with our findings showing IL-10 is associated with increased CAR; however, it is notable that TNFα was not associated with the CAR in our study, suggesting our participants were experiencing less global inflammation. Other studies have identified that TNFα is associated with vulnerability to cognitive and behavioral impairment after sleep deprivation [42], and so either by cognitive modulation or some bidirectional relationship, our participants avoided this additional inflammation. 

Vascular endothelial growth factor A (VEGF-A) is an angiogenic protein and neurotrophic factor [43]. Increased VEGF-A expression has been found in the pre-frontal cortex of mice who undergo chronic restraint stress [44]. Increased levels of plasma VEGF have also been reported in women exposed to prolonged psychosocial stress [45].

Fibroblast growth factor 2 (FGF-2) is a neurotrophic protein with an association to stress in previous studies. Stress-induced glucocorticoids were shown to reduce transcription of FGF-2 in rodent models [46] which is in line with the negative relationship seen in our data. Additionally, higher FGF-2 may play a neuroprotective role following stress [47].

Epidermal growth factor (EGF) is a neurotrophic factor with a relationship to stress that is less well defined [48] but was significant in our study, which may reflect our population and study design compared to previous findings. This novel relationship warrants further investigation. 

Like the CAR and stress, these same cytokines of significance have also been shown to be part of the pathogenesis of several diseases, particularly depression. CXCL1 and VEGF have been shown to contribute to chronic stress-induced depression in rodent models [49,50]. Likewise, VEGF levels were significantly increased in individuals with clinical depression compared with healthy controls [51]. VEGF has also been shown to be a key player in modulation of the sleep/depression relationship, via changes in blood–brain barrier permeability [42]. Interestingly, VEGF-induced angiogenesis in the hippocampus has been shown to be essential for the efficacy of antidepressant medications [43], suggesting a complex role for VEGF in depression. Reduced levels of EGF have been implicated in depression, but EGF levels were not predictive of depression severity [48]. FGF-2 levels were associated with decreased depression-like behaviors in chronic unpredictable stress in mice [52] and FGF-2 administration showed antidepressant-like effects, reducing depression-like behaviors in rodent models [53]. In humans, FGF-2 reactivity predicted anxiety, depression, and stress over the course of the COVID-19 pandemic [54]. Our results may represent the mechanism by which stress induces depression through the signaling of these cytokines, but further study is needed. 

These cytokines have also demonstrated involvement in other psychological disorders. Increased levels of IL-6 were found in patients with PTSD [55]. The same study also identified decreased IL-10 levels in PTSD patients [55]. EGF was found to be negatively correlated with cognitive disfunction in Parkinson’s disease, suggesting a neuroprotective effect [56]. While FGF-2 has been identified for potential use as a biomarker for anxiety and trauma [57]. 

IL-10 balance is crucial as both high and low levels are seen in pathological inflammatory states such as IBD. Decreased levels of IL-10 have been shown in asthma and psoriasis. Higher levels of IL-10 were seen in COVID-19 patients who did not develop long-term sequelae post infection compared to those who did [58]. 

Previous research has indicated that failure to terminate the stress response properly may lead to depressive behaviors; however, this maladaptive response only occurs in a subset of individuals, and the majority are resilient to these effects [59]. EGF, CXCL1, VEGF-A, FGF-2, and IL-6 have all been correlated with stress resilience in prior studies [60]. Our results lend credence to the hypothesis that glucocorticoids serve a dual function that reduces inflammation in the short term but also primes the body to respond to pathogens [61]. Additionally, our results are similar to those found in a meta-analysis of stressor types and immune function showing that brief naturalistic-type stressors tended to cause increases in IL-6 and IL-10 with concomitant decreases in IFNγ [16].

While we did find significant associations between the CAR and several cytokines, we did not find associations with the majority of cytokines included in our panel (IL-1α, IL-1RA, IL-8, IL-15, IL-18, MCP-1, PDGF-AA, TGF, TNFα, CX1CL3, G-CSF, IFNγ, and IL-1β). Our study may have been underpowered to detect associations between the CAR and these cytokines if they exist. However, the CAR is only a single facet of the HPA axis and these cytokines may be under alternative regulation.

To our knowledge, this is the first documentation of such rapid changes in the CAR between days, and so our explanations of this novel phenomenon are inherently speculative. Based on the pattern of CAR exhibited by our participants, it is possible that this may represent induction of and recovery from psychological burnout. On Days 1 and 2, the average CAR is normal to slightly above normal. On Day 3, the CAR is decreased or blunted (Figure 2). Previous findings have associated a blunted CAR with burnout, depression, and self-reported high levels of psychological stress [31,62]. On Day 4, however, the cohort CAR returns to the normal range. The return-to-normal range suggests successful stress inoculation [20]. Other studies have found that when participants appraise the demands of the coming day to be greater than the resources they possess, they subsequently demonstrate a blunted CAR [63], essentially reflecting a state of burnout. Previous research has shown that cognitive processes and emotional attentional control influences diurnal cortisol secretion patterns [64], suggesting that the resilience and emotional intelligence building benefits associated with this training [19,22,65] may be responsible for these shifts in the CAR. There may also be a neuroendocrine feedback component to the observed findings. Cortisol participates in an autoregulatory feedback loop, inhibiting ACTH release, which reduces the stimulus for the adrenal gland to secrete cortisol [66]. On Day 2, the average CAR was slightly above 80% (Figure 2), which is the threshold for what is considered normal. This higher-than-typical level of cortisol may significantly impact ACTH release, leading to the reduced response seen on Day 3. By extension, the low CAR on Day 3 would provide very little inhibition of ACTH, allowing for recovery of the CAR on Day 4. However, regulation of the CAR is multifactorial and therefore the mechanism behind our findings is likely multifactorial as well [66]. 

Military and medical professionals are exposed to intensely stressful situations by the nature of their work. However, personal and public safety depends on these professionals not being cognitively encumbered by their stressors. Previous findings investigating stress mitigation in military personnel suggest that mindfulness training and simulations are most effective [67]. Both mindfulness and simulation are components of this training, which further supports their use in military education. The literature investigating targeted interventions for stress and burnout in medical professionals has been less fruitful [68], but logically, mindfulness and simulation training are appropriate avenues for investigation. This research adds support to the notion that realistic stressor-specific training and mindfulness skills should be an integral component of medical and military education. Our data also have implications for other factors of health, as a chronically elevated CAR is associated with increased risk of cardiovascular mortality [69]. This relationship begins early; investigations of diurnal cortisol rhythms in children showed that childhood cortisol dysregulation was associated with increased risk for cardiovascular disease and allergies in adulthood [70]. The exact mechanisms by which cortisol and CAR dysregulation contribute to cardiovascular disease are not known. However, glucocorticoid receptors are found within the myocardium and throughout the vasculature and may cause changes in contractility and fibrosis [69]. Additionally, downstream effects of cortisol on lipid metabolism and catecholamine secretion may promote hyperlipidemia and hypertension, known contributors to cardiovascular disease and stroke risk [69]. 

While our findings indicate relationships between the CAR and certain cytokines, other investigations on stress and CAR did not find associations with these cytokines [71]. This is likely due to the difference in our stressors, and the fact that most laboratory studies on human stress utilize acute time-limited stressors such as public speaking and mental arithmetic [16]. Intensive Surgical Skills Week is orders of magnitude more stressful than these laboratory tasks and our results are more consistent with immune response patterns seen in brief naturalistic stressors [16]. Additionally, it is worth noting that the increase in effect size when considering cortisol’s effects on a lag is novel and other studies may benefit from considering the delayed effects of steroid signaling. 

Our study further emphasizes the connection between glucocorticoids, CAR, and the hippocampus. Prior studies have related blunted CARs with decreased hippocampal volume [71] and patients with retrograde amnesia do not mount CARs [11]. Directionality is unclear but the combination of a blunted CAR and reduced hippocampal volume has been identified as a risk factor for depression [72]. Low hippocampal volumes are also seen in many pathologies including Alzheimer’s disease, PTSD, Type 2 DM, epilepsy, and Cushing’s disease [73,74], which has implications for the long-term health of those experiencing high levels of stress. Glucocorticoids are proven to be neurotoxic at high levels, and the hippocampus is especially susceptible to neuronal apoptosis [75], suggesting chronic stress may contribute to hippocampal atrophy. The hippocampus is also vulnerable to vascular insults from head injury and hypertension [73], which could then lead to cortisol dysregulation and subsequent toxicity. Our results regarding IFNγ are also relevant to the hippocampal–glucocorticoid relationship as IFNγ has been shown to modulate spatial memory in rodent models of chronic stress [76]. Additionally, a study investigating hippocampal volumes, cortisol levels, and cytokine levels in the elderly found that higher levels of cortisol, IL-6, and TNFα were associated with lower hippocampal volumes [77]. Meanwhile, the same study associated high levels of TNFα and IL-β with decreased hippocampal volume. While we found an association with IL-6 and the CAR, we did not see associations with TNFα or IL-1β, suggesting that TNFα and IL-1β synthesis or release are not related to the CAR in our participants undergoing acute repeated stress. TNFα and IL-1β signaling may represent a more chronic component of stress that contributes to hippocampal atrophy. Studies on antidepressants including SSRIs, SNRIs, and ketamine have shown that all inhibit IL-1β and IL-6’s deleterious effects on hippocampal neurogenesis and apoptosis [78].

### Limitations/Future Directions

This study has several limitations. Firstly, there are many unknowns and possible cofounders regarding our participants including their health history, if injuries were sustained over the week, sleep timing and quality, and the schedule of simulations of each participant during ISSW. Various health conditions, medications, and injuries can all impact the HPA axis and levels of inflammatory markers. 

One of the primary limitations of this study is the absence of a control group of participants who were not exposed to the repeated acute stress induced by the training simulations. While participants served as their own pre-stress controls, without a control group, it is difficult to definitively attribute changes in CAR and immune markers to the stressor rather than to natural physiological fluctuations. The unique structure of the training (e.g., a highly immersive environment, controlled sleep/wake cycles, and isolated barracks) also means that external variables such as sleep quality, diet, and social interactions were strictly controlled, which may not reflect typical daily life. Including a control group in future studies, ideally under matched environmental conditions but without the stress-inducing component, would allow for clearer interpretation of how CAR and cytokine responses are specifically influenced by acute stress. Additionally, without control data from participants in a non-stress scenario, it is difficult to establish whether the blunting and recovery observed in CAR reflects a generalized response to any intensive regimen or is unique to hyper-realistic stress scenarios. 

Furthermore, this study did not perform a gender-specific analysis, which limits its generalizability, particularly in light of existing research indicating that males and females can exhibit differential CAR patterns and immune responses under stress [11]. Variations in hormonal cycles, particularly in females, have been shown to affect cortisol dynamics and cytokine levels [79]. This omission could mean that our findings primarily reflect the average response across genders rather than identifying unique gender-specific response patterns. Studies have found that females often exhibit stronger immune responses but also face higher risks of autoimmune reactions under stress [11]. Consequently, the lack of stratification by gender may obscure meaningful differences in stress adaptation and immune function. We acknowledge these potential discrepancies, as gender-specific data could yield insights into tailoring stress management techniques in high-stress environments like military or medical fields. Future research with gender-stratified sampling or analyses could significantly improve the accuracy of our findings. 

In future studies investigating CAR, best practices suggest collecting three samples in order to calculate the area under the curve for the response, as well as electronically verifying participant wake times due to the possible impact on CAR magnitude [80,81]. Therefore, these findings should be interpreted with caution. 

Due to the nature of our study design, sampling on Day 5 after the students were allowed to leave the controlled environment would be of little benefit. Future studies should consider the use of post stressor sampling to observe the recovery period of the stress response. Additionally, TNFα, IL-1β, INFγ, CX3CL1, and G-CSF were only analyzed in the 2023 cohort, which limits the strength of the interpretations that can be drawn from those markers. Our sample is also rather homogenous, which may limit the generalizability of our findings. The type of stress and stress training experienced at ISSW is unique compared to stress experienced by the general non-military or first-responder population. However, the health and impact of stress on military and medical personnel is of vital importance to the optimal functioning of these individuals and merits research despite the specialized population applications and limited generalizability. 

While stimulated whole salivary collection is noninvasive and an accurate reflection of serum levels, it is not without its drawbacks, as there is a lack of standardization of saliva flow rates as well as individual differences in storage and shipment of samples that could impact biomarker analysis. Furthermore, our results are taken from two time points in the morning; measurement of these cytokines throughout the course of a day may elucidate different patterns and provide more complete explanations of these interactions. 

Our data analysis handled participant data in aggregate, and further work is required to identify individual differences in stress responses and immune function, particularly to identify factors associated with stress resilience. Further investigation may lead to the ability to identify individuals who are at increased risk of developing pathology in response to high levels of stress, and potential targets for the prevention and treatment of a wide array of stress-related pathologies. In addition, our power analysis indicates a 72% chance to detect a difference of one-half of a standard deviation, which is below the typical threshold of 80%. So, our study may be underpowered to detect small-to-moderate effects, and future studies would benefit from a larger sample. 

Due to the unique, temporal, and exploratory nature of our study our analysis included many cytokines; this led to a large number of samples and complex analysis and interpretation. Future endeavors may reduce this complexity by more targeted selection of cytokines for investigation. Due to the exploratory nature of the study, our proposed explanation for the observed CAR pattern is inherently speculative, and further investigation is needed to elucidate the exact mechanism(s) behind our findings. 

Of note, other classically inflammatory cytokines with roles in pathology including IL-1β and TNFα were nonsignificant in our study, though both were only analyzed in half of our cohort. These differences are likely attributable to the nature of our population and the training itself, but further study is needed to clarify these observations. 

## 5. Conclusions

We were able to identify a novel pattern of cortisol awakening response in the context of repeated acute stress in healthy young adults (Figure 2). We suggest this dampening and then recovery of the CAR represents induction of and recovery from psychological burnout in our participants and adds support for simulation training as a component of medical and military education.

Our results also show broad patterns in salivary cytokine signaling in the post wake period during repeated acute stress. As is consistent with the conceptualization of cortisol’s immune suppression properties, interleukins decreased in concentration between Time 1 and Time 2 (Figure 3), while cortisol increased from Time 1 to Time 2 (Figure 2). Many cytokines showed changes in salivary concentrations from T1 to T2 (Figure 3, Figure 4 and Figure 5), suggesting there may be daily cycles to immune system functions as well. The relationship between these cytokine changes and the CAR is unclear. Our study design does not allow for causal relationships to be drawn, and no cytokines showed the same significant blunting as the CAR. Even when considering the lagged nature of cortisol, CAR is only moderately associated with a subset of the cytokines in our study. As is consistent with the current literature, immune system function is multifactorial and the CAR is not the only manifestation of stress response. Our study found significant interactions between stress and cytokines implicated in multiple pathological processes including depression and PTSD. Signaling through these pathways may represent how stress contributes to the pathogenesis of these disorders as well as other conditions associated with high levels of stress including susceptibility to infection.

Our results shed light on the impact of repeated acute stress on the CAR and the immune system. Our study also begins to fill knowledge gaps within the field due to our unique study design which allows us to ethically apply stressors to human subjects in a controlled environment. Many professionals are unable to avoid stress in the context of their work, and this study is a key step to help us identify individuals who are at risk of developing pathology such as depression and cardiovascular disease due to stress exposure. Our work also paves the way forward for identifying targets for preventing and managing stress-related pathology including career-specific simulation training.

## Figures and Tables

**Table 1:** Sample demographic information.

Sample Characteristic		n	%	Mean	SD
Gender					
	Male	51	63.75%		
	Female	29	36.25%		
Race					
	White	70	87.5%		
	Asian	4	5%		
	Black	3	3.75%		
	Multiracial	3	3.75%		
Ethnicity					
	Hispanic	5	6.25%		
	Non-Hispanic	75	93.75%		
Previous Military Service					
	Yes	9	11.25%		
	No	71	88.75%		
Previous Combat Experience					
	Yes	0	0%		
	No	80	100%		
Age (years)				26.63	2.18

**Table 2:** Significant Pearson correlations between CAR and mean daily salivary cytokine levels during repeated acute stress with α = 0.05. Effect size is given by r^2^.

Cytokine	r	r^2^	*p*-Value
CXCL1	0.2	0.04	0.0005
IL-6	0.13	0.017	0.02
IL-10	0.14	0.02	0.02
VEGF-A	0.17	0.029	0.003

**Table 3:** Significant Pearson correlations between Day 2 CAR and Day 3 Time 1 salivary cytokine levels during repeated acute stress with α = 0.05. Effect size is given by r^2^.

Cytokine (Day 3 T1)	r	r^2^	*p*-Value
CXCL1	0.41	0.17	0.0002
IL-6	0.38	0.14	0.0006
IL-10	0.3	0.09	0.008
VEGF-A	0.41	0.17	0.0002

**Table 4:** Significant Pearson correlations between Day 3 CAR and Day 4 Time 1 salivary cytokines during repeated acute stress with α = 0.05. Effect size is given by r^2^.

Cytokine (Day 4 T1)	r	r^2^	*p*-Value
EGF	0.27	0.073	0.02
FGF-2	−0.29	0.084	0.01

## Data Availability

Due to the large and complex nature of the dataset, the raw data supporting the conclusions of this article will be made available by the authors on request.

## Supplementary Materials

The following supporting information can be downloaded at https://www.mdpi.com/article/10.3390/biomedicines12112519/s1; Tables S1–S38: Cytokine descriptive statistics, and cytokine Tukey’s test results.
