# Supplementary material for: A Prospective Cohort Study on the Effects of Repeated Acute Stress on Cortisol Awakening Response and Immune Function in Military Medical Students
Source: Biomedicines. 2024 Nov 4;12(11):2519. doi: 10.3390/biomedicines12112519 (PMC11592205; doi:10.3390/biomedicines12112519)
Supplement: Supplementary file 1 [file biomedicines-12-02519-s001.zip › Supplemental Tables.pdf]

EGF

Table S1. Descriptive statistics for daily cohort salivary levels of EGF at Time 1 (waking) and Time 2 (30 minutes post waking) during repeated acute stress.

Day	N, samples	Range (pg/mL)	Mean pg/mL	Std Deviation (pg/mL)	SEM (pg/mL)
Day 1	79	8085	1689	1391	156.5
	76	3572	765.1	685.3	78.61
Day 2	78	4534	1495	865.1	97.96
	79	3459	661.6	587	66.05
Day 3	78	21070	1842	2458	278.3
	79	6266	717.4	793.8	89.31
Day 4	79	3194	1416	616.9	69.41
	75	1984	585.9	364.2	42.06

Table S2. Results of Tukey's multiple comparison tests between cohort average salivary EGF levels at each measurement point Days 1-4, with sampling at Time 1 (waking) and Time 2 (30 minutes post waking) each day during repeated acute stress.

Tukey's multiple comparisons test	Mean Diff.	95.00% CI of diff.	Summary	Adjusted P Value
D1 T1 vs. D1 T2	962.8	574.0 to 1352	****	<0.0001
D1 T1 vs. D2 T1	188.9	-282.8 to 660.6	ns	0.9146
D1 T1 vs. D2 T2	1027	603.4 to 1451	****	<0.0001
D1 T1 vs. D3 T1	-143.3	-916.6 to 630.0	ns	0.9991
D1 T1 vs. D3 T2	971.2	533.5 to 1409	****	<0.0001
D1 T1 vs. D4 T1	272.9	-177.5 to 723.4	ns	0.5636
D1 T1 vs. D4 T1	1132	657.2 to 1607	****	<0.0001
D1 T2 vs. D2 T1	-756.2	-1012 to -500.8	****	<0.0001
D1 T2 vs. D2 T2	90.45	-50.47 to 231.4	ns	0.4879
D1 T2 vs. D3 T1	-1118	-1868 to -368.7	***	0.0004
D1 T2 vs. D3 T2	38.23	-128.7 to 205.1	ns	0.9963
D1 T2 vs. D4 T1	-656.4	-912.8 to -400.1	****	<0.0001
D1 T2 vs. D4 T1	185.8	8.074 to 363.6	*	0.0341
D2 T1 vs. D2 T2	837	612.2 to 1062	****	<0.0001
D2 T1 vs. D3 T1	-334.1	-1078 to 409.4	ns	0.8542
D2 T1 vs. D3 T2	776.5	527.8 to 1025	****	<0.0001
D2 T1 vs. D4 T1	79.37	-214.0 to 372.7	ns	0.9899
D2 T1 vs. D4 T1	931.3	673.5 to 1189	****	<0.0001
D2 T2 vs. D3 T1	-1176	-1945 to -407.4	***	0.0002
D2 T2 vs. D3 T2	-55.79	-233.1 to 121.5	ns	0.976
D2 T2 vs. D4 T1	-754.1	-999.0 to -509.1	****	<0.0001
D2 T2 vs. D4 T1	93.49	-52.99 to 240.0	ns	0.495
D3 T1 vs. D3 T2	1121	501.6 to 1740	****	<0.0001
D3 T1 vs. D4 T1	424.8	-389.1 to 1239	ns	0.733
D3 T1 vs. D4 T1	1287	433.5 to 2141	***	0.0003
D3 T2 vs. D4 T1	-698.3	-982.1 to -414.4	****	<0.0001
D3 T2 vs. D4 T1	146.2	-78.72 to 371.1	ns	0.4709
D4 T1 vs. D4 T1	832.4	636.0 to 1029	****	<0.0001

FGF-2

Table S3. Descriptive statistics for daily cohort salivary levels of FGF-2 at Time 1 (waking) and Time 2 (30 minutes post waking) during repeated acute stress.

Day	N, samples	Range (pg/mL)	Mean pg/mL	Std Deviation (pg/mL)	SEM (pg/mL)
Day 1	79	202.4	34.34	28.48	3.204
	76	97.04	21.12	17.38	1.994
Day 2	78	290.4	24.08	33.19	3.758
	79	117.1	12.10	16.79	1.889
Day 3	78	320.9	31.91	36.35	4.115
	79	106.8	19.91	15.98	1.798
Day 4	79	131.3	23.95	20.37	2.291
	75	166.4	20.15	28.05	3.239

Table S4. Results of Tukey's multiple comparison tests between cohort average salivary FGF-2 levels at each measurement point Days 1-4, with sampling at Time 1 (waking) and Time 2 (30 minutes post waking) each day during repeated acute stress.

Tukey's multiple comparisons test	Mean Diff.	95.00% CI of diff.	Summary	Adjusted P Value
D1 T1 vs. D1 T2	13.6	5.713 to 21.50	****	<0.0001
D1 T1 vs. D2 T1	10.44	3.300 to 17.58	***	0.0005
D1 T1 vs. D2 T2	22.23	14.41 to 30.05	****	<0.0001
D1 T1 vs. D3 T1	2.638	-4.954 to 10.23	ns	0.9585
D1 T1 vs. D3 T2	14.42	5.914 to 22.93	****	<0.0001
D1 T1 vs. D4 T1	10.39	4.211 to 16.57	****	<0.0001
D1 T1 vs. D4 T2	14.32	3.195 to 25.44	**	0.0034
D1 T2 vs. D2 T1	-3.391	-13.05 to 6.264	ns	0.9558
D1 T2 vs. D2 T2	8.574	2.544 to 14.60	***	0.0008
D1 T2 vs. D3 T1	-10.98	-21.81 to -0.1525	*	0.0445
D1 T2 vs. D3 T2	0.9061	-4.190 to 6.002	ns	0.9993
D1 T2 vs. D4 T1	-2.516	-8.244 to 3.213	ns	0.8682
D1 T2 vs. D4 T2	1.178	-8.649 to 11.00	ns	>0.9999
D2 T1 vs. D2 T2	12.02	4.074 to 19.96	***	0.0003
D2 T1 vs. D3 T1	-7.595	-12.08 to -3.108	****	<0.0001
D2 T1 vs. D3 T2	4.334	-4.738 to 13.41	ns	0.8112
D2 T1 vs. D4 T1	0.176	-7.692 to 8.044	ns	>0.9999
D2 T1 vs. D4 T2	3.915	-8.531 to 16.36	ns	0.9757
D2 T2 vs. D3 T1	-19.65	-28.73 to -10.57	****	<0.0001
D2 T2 vs. D3 T2	-7.809	-12.76 to -2.862	***	0.0001
D2 T2 vs. D4 T1	-11.84	-17.92 to -5.767	****	<0.0001
D2 T2 vs. D4 T2	-7.674	-16.58 to 1.234	ns	0.143
D3 T1 vs. D3 T2	12	2.008 to 21.99	**	0.008
D3 T1 vs. D4 T1	7.766	-0.4721 to 16.00	ns	0.0791
D3 T1 vs. D4 T2	12.23	-1.096 to 25.56	ns	0.0954
D3 T2 vs. D4 T1	-4.032	-10.13 to 2.065	ns	0.4497
D3 T2 vs. D4 T2	0.228	-8.343 to 8.799	ns	>0.9999
D4 T1 vs. D4 T2	3.71	-5.949 to 13.37	ns	0.93

CXCL1

Table S5. Descriptive statistics for daily cohort salivary levels of CXCL1 at Time 1 (waking) and Time 2 (30 minutes post waking) during repeated acute stress.

Day	N, samples	Range (pg/mL)	Mean (pg/mL)	Std Deviation (pg/mL)	SEM (pg/mL)
Day 1	79	754.7	41.26	111.1	12.5
	76	302.3	29.29	62.37	7.155
Day 2	78	750.2	26.93	90.45	10.24
	79	419.5	41.56	81.29	9.145
Day 3	78	418.4	24.15	58.85	6.663
	79	541	45.17	97.08	10.92
Day 4	78	196.2	17.64	35.65	4.037
	75	302.4	35.42	64.94	7.499

Table S6. Results of Tukey's multiple comparison tests between cohort average salivary CXCL1 levels at each measurement point days 1-4, with sampling at Time 1 (waking) and Time 2 (30 minutes post waking) each day during repeated acute stress.

Tukey's multiple comparisons test	Mean Diff.	95.00% CI of diff.	Summary	Adjusted P Value
D1 T1 vs. D1 T2	1.785	-22.12 to 25.69	ns	>0.9999
D1 T1 vs. D2 T1	14.75	-13.04 to 42.53	ns	0.7163
D1 T1 vs. D2 T2	-0.3004	-39.44 to 38.84	n	>0.9999
D1 T1 vs. D3 T1	17.54	-16.24 to 51.32	ns	0.7384
D1 T1 vs. D3 T2	-3.91	-42.31 to 34.49	ns	>0.9999
D1 T1 vs. D4 T1	23.96	-10.39 to 58.31	ns	0.3792
D1 T1 vs. D4 T2	7.254	-29.32 to 43.82	ns	0.9985
D1 T2 vs. D2 T1	5.858	-21.05 to 32.76	ns	0.9973
D1 T2 vs. D2 T2	-10.04	-37.92 to 17.84	ns	0.9498
D1 T2 vs. D3 T1	6.723	-16.14 to 29.59	ns	0.9835
D1 T2 vs. D3 T2	-15.58	-49.12 to 17.95	ns	0.8311
D1 T2 vs. D4 T1	12.73	-8.878 to 34.34	ns	0.597
D1 T2 vs. D4 T2	-4.237	-28.73 to 20.26	ns	0.9994
D2 T1 vs. D2 T2	-15.1	-50.48 to 20.28	ns	0.8846
D2 T1 vs. D3 T1	2.853	-16.12 to 21.82	ns	0.9998
D2 T1 vs. D3 T2	-18.79	-55.05 to 17.48	ns	0.7406
D2 T1 vs. D4 T1	9.509	-17.20 to 36.22	ns	0.9528
D2 T1 vs. D4 T2	-8.599	-39.98 to 22.78	ns	0.989
D2 T2 vs. D3 T1	17.92	-6.831 to 42.67	ns	0.3313
D2 T2 vs. D3 T2	-3.61	-24.11 to 16.89	ns	0.9993
D2 T2 vs. D4 T1	24.35	-0.6343 to 49.34	ns	0.0615
D2 T2 vs. D4 T2	6.893	-11.73 to 25.52	ns	0.9419
D3 T1 vs. D3 T2	-21.58	-49.53 to 6.368	ns	0.2534
D3 T1 vs. D4 T1	6.559	-6.690 to 19.81	ns	0.7816
D3 T1 vs. D4 T2	-12.17	-31.49 to 7.146	ns	0.5114
D3 T2 vs. D4 T1	27.63	0.7876 to 54.47	*	0.0391
D3 T2 vs. D4 T2	10.17	-10.84 to 31.18	ns	0.8
D4 T1 vs. D4 T2	-17.64	-35.45 to 0.1707	ns	0.0541

IL-1α

Table S7. Descriptive statistics for daily cohort salivary levels of IL-1α at Time 1 (waking) and Time 2 (30 minutes post waking) during repeated acute stress.

Day	N, Samples	Range (pg/mL)	Mean (pg/mL)	Std Deviation (pg/mL)	SEM (pg/mL)
Day 1	78	48115	9694	10218	1157
	76	19462	3849	3198	366.9
Day 2	78	20308	6612	4133	468
	79	23764	2972	3049	343
Day 3	77	62533	9140	9859	1124
	79	17435	3344	3174	357.1
Day 4	79	11738	5288	2751	309.5
	75	6534	2281	1488	171.8

Table S8. Results of Tukey's multiple comparison tests between cohort average salivary IL-1α levels at each measurement point Days 1-4, with sampling at Time 1 (waking) and Time 2 (30 minutes post waking) each day during repeated acute stress.

Tukey's multiple comparisons test	Mean Diff.	95.00% CI of diff.	Summary	Adjusted P Value
D1 T1 vs. D1 T2	6063	2918 to 9208	****	<0.0001
D1 T1 vs. D2 T1	2550	-858.5 to 5959	ns	0.2899
D1 T1 vs. D2 T2	6675	3371 to 9978	****	<0.0001
D1 T1 vs. D3 T1	265.3	-3033 to 3564	ns	>0.9999
D1 T1 vs. D3 T2	6136	3055 to 9217	****	<0.0001
D1 T1 vs. D4 T1	4039	905.3 to 7172	**	0.0033
D1 T1 vs. D4 T2	7341	3927 to 10755	****	<0.0001
D1 T2 vs. D2 T1	-3337	-4964 to -1709	****	<0.0001
D1 T2 vs. D2 T2	793.6	94.21 to 1493	*	0.0153
D1 T2 vs. D3 T1	-5614	-8759 to -2470	****	<0.0001
D1 T2 vs. D3 T2	258.1	-743.0 to 1259	ns	0.9923
D1 T2 vs. D4 T1	-1768	-2900 to -635.9	***	0.0002
D1 T2 vs. D4 T2	1282	576.3 to 1988	****	<0.0001
D2 T1 vs. D2 T2	4061	2664 to 5458	****	<0.0001
D2 T1 vs. D3 T1	-2032	-5148 to 1084	ns	0.4659
D2 T1 vs. D3 T2	3542	1864 to 5220	****	<0.0001
D2 T1 vs. D4 T1	1466	218.4 to 2715	*	0.0104
D2 T1 vs. D4 T2	4590	3053 to 6128	****	<0.0001
D2 T2 vs. D3 T1	-6131	-9313 to -2949	****	<0.0001
D2 T2 vs. D3 T2	-538.3	-1392 to 315.4	ns	0.5113
D2 T2 vs. D4 T1	-2636	-3630 to -1641	****	<0.0001
D2 T2 vs. D4 T2	490.8	43.06 to 938.6	*	0.0218
D3 T1 vs. D3 T2	5612	2820 to 8404	****	<0.0001
D3 T1 vs. D4 T1	3459	349.2 to 6568	*	0.0188
D3 T1 vs. D4 T2	6816	3528 to 10104	****	<0.0001
D3 T2 vs. D4 T1	-2097	-3176 to -1019	****	<0.0001
D3 T2 vs. D4 T2	1077	316.3 to 1838	***	0.0009
D4 T1 vs. D4 T2	3189	2229 to 4149	****	<0.0001

IL-1RA

Table S9. Descriptive statistics for daily cohort salivary levels of IL-1RA at Time 1 (waking) and Time 2 (30 minutes post waking) during repeated acute stress.

Day	N, samples	Range (pg/mL)	Mean (pg/mL)	Std Deviation (pg/mL)	SEM (pg/mL)
Day 1	79	8786	3640	1417	159.4
	76	5543	2827	1021	117.2
Day 2	78	3099	2919	692.2	78.37
	79	3940	2322	741.1	83.38
Day 3	78	4678	3409	1006	113.9
	79	4245	2578	833.1	93.73
Day 4	79	3974	2690	978.7	110.1
	75	2934	1978	688.1	79.46

Table S10. Results of Tukey's multiple comparison tests between cohort average salivary IL-1RA levels at each measurement point Days 1-4, with sampling at Time 1 (waking) and Time 2 (30 minutes post waking) each day during repeated acute stress.

Tukey's multiple comparisons test	Mean Diff.	95.00% CI of diff.	Summary	Adjusted P Value
D1 T1 vs. D1 T2	822.8	475.0 to 1171	****	<0.0001
D1 T1 vs. D2 T1	724.4	168.4 to 1280	**	0.0028
D1 T1 vs. D2 T2	1318	771.3 to 1864	****	<0.0001
D1 T1 vs. D3 T1	252.7	-164.4 to 669.9	ns	0.5635
D1 T1 vs. D3 T2	1062	663.8 to 1460	****	<0.0001
D1 T1 vs. D4 T1	949.6	422.7 to 1476	****	<0.0001
D1 T1 vs. D4 T2	1665	1125 to 2204	****	<0.0001
D1 T2 vs. D2 T1	-76.34	-560.3 to 407.6	ns	0.9997
D1 T2 vs. D2 T2	508.7	84.34 to 933.1	**	0.0082
D1 T2 vs. D3 T1	-609.3	-978.0 to -240.7	****	<0.0001
D1 T2 vs. D3 T2	263.8	-53.52 to 581.0	ns	0.1752
D1 T2 vs. D4 T1	149.5	-343.0 to 641.9	ns	0.9803
D1 T2 vs. D4 T2	849.3	421.6 to 1277	****	<0.0001
D2 T1 vs. D2 T2	618.6	317.0 to 920.2	****	<0.0001
D2 T1 vs. D3 T1	-470.2	-828.5 to -111.9	**	0.0026
D2 T1 vs. D3 T2	346.4	-11.77 to 704.5	ns	0.0653
D2 T1 vs. D4 T1	220	-28.72 to 468.6	ns	0.1219
D2 T1 vs. D4 T2	918.1	687.1 to 1149	****	<0.0001
D2 T2 vs. D3 T1	-1079	-1506 to -651.9	****	<0.0001
D2 T2 vs. D3 T2	-255.5	-608.3 to 97.35	ns	0.3315
D2 T2 vs. D4 T1	-368	-783.7 to 47.70	ns	0.1214
D2 T2 vs. D4 T2	336	23.88 to 648.1	*	0.0259
D3 T1 vs. D3 T2	815	548.5 to 1082	****	<0.0001
D3 T1 vs. D4 T1	700.6	334.9 to 1066	****	<0.0001
D3 T1 vs. D4 T2	1404	1064 to 1745	****	<0.0001
D3 T2 vs. D4 T1	-112.5	-493.0 to 268.0	ns	0.9831
D3 T2 vs. D4 T2	593	284.8 to 901.2	****	<0.0001
D4 T1 vs. D4 T2	699.5	428.8 to 970.1	****	<0.0001

IL-6

Table S11. Descriptive statistics for daily cohort salivary levels of IL-6 at Time 1 (waking) and Time 2 (30 minutes post waking) during repeated acute stress.

Day	N, samples	Range (pg/mL)	Mean (pg/mL)	Std Deviation (pg/mL)	SEM (pg/mL)
Day 1	79	31.2	4.434	4.737	0.533
	76	9.81	2.175	1.786	0.2048
Day 2	78	28.87	3.281	4.999	0.566
	78	10.48	1.47	1.611	0.1825
Day 3	78	14.39	2.666	2.81	0.3181
	78	10.46	1.636	1.738	0.1968
Day 4	79	13.91	2.69	2.956	0.3326
	75	8.22	1.254	1.352	0.1561

Table S12. Results of Tukey's multiple comparison tests between cohort average salivary IL-6 levels at each measurement point Days 1-4, with sampling at Time 1 (waking) and Time 2 (30 minutes post waking) each day during repeated acute stress.

Tukey's multiple comparisons test	Mean Diff.	95.00% CI of diff.	Summary	Adjusted P Value
D1 T1 vs. D1 T2	2.196	0.5906 to 3.801	**	0.0014
D1 T1 vs. D2 T1	1.183	-0.7879 to 3.154	ns	0.575
D1 T1 vs. D2 T2	2.883	1.555 to 4.211	****	<0.0001
D1 T1 vs. D3 T1	1.779	0.4179 to 3.140	**	0.0027
D1 T1 vs. D3 T2	2.84	1.443 to 4.237	****	<0.0001
D1 T1 vs. D4 T1	1.744	0.1512 to 3.336	*	0.0219
D1 T1 vs. D4 T2	3.279	1.701 to 4.857	****	<0.0001
D1 T2 vs. D2 T1	-1.039	-2.765 to 0.6864	ns	0.5698
D1 T2 vs. D2 T2	0.7761	0.1104 to 1.442	*	0.0114
D1 T2 vs. D3 T1	-0.5099	-1.454 to 0.4341	ns	0.6968
D1 T2 vs. D3 T2	0.5692	-0.1476 to 1.286	ns	0.2217
D1 T2 vs. D4 T1	-0.5453	-1.499 to 0.4088	ns	0.6339
D1 T2 vs. D4 T2	0.9108	0.1782 to 1.643	**	0.0053
D2 T1 vs. D2 T2	1.855	0.2504 to 3.459	*	0.0124
D2 T1 vs. D3 T1	0.6109	-0.9986 to 2.220	ns	0.9344
D2 T1 vs. D3 T2	1.669	-0.01593 to 3.355	ns	0.054
D2 T1 vs. D4 T1	0.5709	-1.125 to 2.267	ns	0.9651
D2 T1 vs. D4 T2	2.061	0.1963 to 3.926	*	0.02
D2 T2 vs. D3 T1	-1.209	-1.909 to -0.5080	****	<0.0001
D2 T2 vs. D3 T2	-0.1735	-0.5163 to 0.1693	ns	0.762
D2 T2 vs. D4 T1	-1.234	-2.145 to -0.3237	**	0.0016
D2 T2 vs. D4 T2	0.2454	-0.2082 to 0.6990	ns	0.6947
D3 T1 vs. D3 T2	1.057	0.3540 to 1.759	***	0.0003
D3 T1 vs. D4 T1	-0.03256	-0.9874 to 0.9223	ns	>0.9999
D3 T1 vs. D4 T2	1.509	0.6461 to 2.373	****	<0.0001
D3 T2 vs. D4 T1	-1.082	-1.946 to -0.2185	**	0.0048
D3 T2 vs. D4 T2	0.4034	-0.07759 to 0.8843	ns	0.1666
D4 T1 vs. D4 T2	1.505	0.6938 to 2.316	****	<0.0001

IL-8

Table S13. Descriptive statistics for daily cohort salivary levels of IL-8 at Time 1 (waking) and Time 2 (30 minutes post waking) during repeated acute stress.

Day	N, samples	Range (pg/mL)	Mean (pg/mL)	Std Deviation (pg/mL)	SEM (pg/mL)
Day 1	79	4010	1575	835.7	94.02
	76	3278	662.1	529.7	60.76
Day 2	78	4786	1465	772.6	87.48
	79	3311	489.5	455.2	51.22
Day 3	78	2854	1429	609	68.96
	79	2200	490.1	375.2	42.21
Day 4	79	6150	1372	923.9	103.9
	75	2454	416	348.3	40.21

Table S14. Results of Tukey's multiple comparison tests between cohort average salivary IL-8 levels at each measurement point Days 1-4, with sampling at Time 1 (waking) and Time 2 (30 minutes post waking) each day during repeated acute stress.

Tukey's multiple comparisons test	Mean Diff.	95.00% CI of diff.	Summary	Adjusted P Value
D1 T1 vs. D1 T2	937.2	662.7 to 1212	****	<0.0001
D1 T1 vs. D2 T1	98.91	-207.2 to 405.0	ns	0.9721
D1 T1 vs. D2 T2	1085	799.1 to 1371	****	<0.0001
D1 T1 vs. D3 T1	157	-91.48 to 405.4	ns	0.5094
D1 T1 vs. D3 T2	1085	816.8 to 1352	****	<0.0001
D1 T1 vs. D4 T1	203.1	-179.5 to 585.6	ns	0.7167
D1 T1 vs. D4 T2	1182	891.8 to 1473	****	<0.0001
D1 T2 vs. D2 T1	-818.3	-1135 to -501.8	****	<0.0001
D1 T2 vs. D2 T2	165.5	-0.08518 to 331.1	ns	0.0502
D1 T2 vs. D3 T1	-780.8	-1004 to -558.0	****	<0.0001
D1 T2 vs. D3 T2	164.4	8.934 to 319.8	*	0.0307
D1 T2 vs. D4 T1	-706.8	-1012 to -401.7	****	<0.0001
D1 T2 vs. D4 T2	248	74.52 to 421.5	***	0.0007
D2 T1 vs. D2 T2	980.7	709.1 to 1252	****	<0.0001
D2 T1 vs. D3 T1	50.52	-163.2 to 264.3	ns	0.9956
D2 T1 vs. D3 T2	978.6	716.1 to 1241	****	<0.0001
D2 T1 vs. D4 T1	98.72	-236.6 to 434.0	ns	0.9835
D2 T1 vs. D4 T2	1050	791.7 to 1309	****	<0.0001
D2 T2 vs. D3 T1	-935.6	-1151 to -719.9	****	<0.0001
D2 T2 vs. D3 T2	-0.6111	-92.22 to 91.00	ns	>0.9999
D2 T2 vs. D4 T1	-882.1	-1204 to -559.7	****	<0.0001
D2 T2 vs. D4 T2	85.16	-4.429 to 174.7	ns	0.0743
D3 T1 vs. D3 T2	934.2	765.8 to 1103	****	<0.0001
D3 T1 vs. D4 T1	43.32	-256.1 to 342.7	ns	0.9998
D3 T1 vs. D4 T2	1017	823.6 to 1210	****	<0.0001
D3 T2 vs. D4 T1	-881.5	-1184 to -579.4	****	<0.0001
D3 T2 vs. D4 T2	88.3	5.666 to 170.9	*	0.0278
D4 T1 vs. D4 T2	975.4	660.0 to 1291	****	<0.0001

IL-10

Table S15. Descriptive statistics for daily cohort salivary levels of IL-10 at Time 1 (waking) and Time 2 (30 minutes post waking) during repeated acute stress.

Day	N, samples	Range (pg/mL)	Mean (pg/mL)	Std Deviation (pg/mL)	SEM (pg/mL)
Day 1	72	22.25	3.765	3.509	0.4135
	70	8.39	2.231	1.757	0.21
Day 2	69	21.52	3.696	4.229	0.5091
	67	12.99	1.717	2.012	0.2458
Day 3	77	16.72	3.44	3.498	0.3987
	74	11.56	2.319	2.39	0.2779
Day 4	74	14.91	3.278	3.448	0.4008
	66	10.99	1.737	1.7	0.2092

Table S16. Results of Tukey's multiple comparison tests between cohort average salivary IL-10 levels at each measurement point Days 1-4, with sampling at Time 1 (waking) and Time 2 (30 minutes post waking) each day during repeated acute stress.

Tukey's multiple comparisons test	Mean Diff.	95.00% CI of diff.	Summary	Adjusted P Value
D1 T1 vs. D1 T2	1.54	0.3595 to 2.720	**	0.003
D1 T1 vs. D2 T1	0.2141	-1.018 to 1.446	ns	0.9993
D1 T1 vs. D2 T2	2.166	0.6272 to 3.705	**	0.001
D1 T1 vs. D3 T1	0.2269	-0.9931 to 1.447	ns	0.999
D1 T1 vs. D3 T2	1.48	0.03051 to 2.929	*	0.042
D1 T1 vs. D4 T1	0.3796	-0.8256 to 1.585	ns	0.975
D1 T1 vs. D4 T2	2.189	0.5553 to 3.822	**	0.0021
D1 T2 vs. D2 T1	-1.479	-2.937 to -0.02138	*	0.0443
D1 T2 vs. D2 T2	0.5683	-0.2277 to 1.364	ns	0.3414
D1 T2 vs. D3 T1	-1.308	-2.307 to -0.3091	**	0.0028
D1 T2 vs. D3 T2	-0.1785	-0.9540 to 0.5970	ns	0.9961
D1 T2 vs. D4 T1	-1.098	-2.298 to 0.1014	ns	0.0965
D1 T2 vs. D4 T2	0.5714	-0.3453 to 1.488	ns	0.517
D2 T1 vs. D2 T2	2.073	0.4046 to 3.741	**	0.0056
D2 T1 vs. D3 T1	-0.01838	-1.104 to 1.068	ns	>0.9999
D2 T1 vs. D3 T2	1.4	-0.2666 to 3.066	ns	0.1638
D2 T1 vs. D4 T1	0.2248	-1.021 to 1.471	ns	0.9992
D2 T1 vs. D4 T2	2.133	0.2551 to 4.011	*	0.0156
D2 T2 vs. D3 T1	-2.045	-3.274 to -0.8156	****	<0.0001
D2 T2 vs. D3 T2	-0.7767	-1.635 to 0.08143	ns	0.1039
D2 T2 vs. D4 T1	-1.814	-3.330 to -0.2991	**	0.0086
D2 T2 vs. D4 T2	-0.06138	-0.9491 to 0.8263	ns	>0.9999
D3 T1 vs. D3 T2	1.23	0.2012 to 2.259	**	0.0086
D3 T1 vs. D4 T1	0.2603	-0.8132 to 1.334	ns	0.9947
D3 T1 vs. D4 T2	2.007	0.5820 to 3.432	**	0.001
D3 T2 vs. D4 T1	-0.9992	-2.278 to 0.2801	ns	0.2388
D3 T2 vs. D4 T2	0.7456	-0.2630 to 1.754	ns	0.3007
D4 T1 vs. D4 T2	1.749	0.2728 to 3.226	**	0.0098

IL-15

Table S17. Descriptive statistics for daily cohort salivary levels of IL-15 at Time 1 (waking) and Time 2 (30 minutes post waking) during repeated acute stress.

Day	N, samples	Range (pg/mL)	Mean (pg/mL)	Std Deviation (pg/mL)	SEM (pg/mL)
Day 1	79	14.21	5.878	2.652	0.2984
	76	7.81	4.048	1.719	0.1972
Day 2	78	26.59	5.144	3.258	0.3689
	79	6.17	3.243	1.41	0.1587
Day 3	78	30.1	6.033	3.762	0.426
	79	7.2	3.845	1.854	0.2086
Day 4	79	10.19	4.495	1.768	0.1989
	75	12.04	3.189	1.721	0.1987

Table 18. Results of Tukey's multiple comparison tests between cohort average salivary IL-15 levels at each measurement point Days 1-4, with sampling at Time 1 (waking) and Time 2 (30 minutes post waking) each day during repeated acute stress.

Tukey's multiple comparisons test	Mean Diff.	95.00% CI of diff.	Summary	Adjusted P Value
D1 T1 vs. D1 T2	1.894	1.128 to 2.659	****	<0.0001
D1 T1 vs. D2 T1	0.7371	-0.1376 to 1.612	ns	0.1627
D1 T1 vs. D2 T2	2.635	1.797 to 3.473	****	<0.0001
D1 T1 vs. D3 T1	-0.146	-1.096 to 0.8038	ns	0.9997
D1 T1 vs. D3 T2	2.032	1.239 to 2.826	****	<0.0001
D1 T1 vs. D4 T1	1.382	0.6123 to 2.152	****	<0.0001
D1 T1 vs. D4 T2	2.849	1.870 to 3.827	****	<0.0001
D1 T2 vs. D2 T1	-1.152	-2.288 to -0.01569	*	0.0446
D1 T2 vs. D2 T2	0.7676	0.2698 to 1.265	***	0.0002
D1 T2 vs. D3 T1	-2.077	-3.276 to -0.8770	****	<0.0001
D1 T2 vs. D3 T2	0.1567	-0.3338 to 0.6473	ns	0.9736
D1 T2 vs. D4 T1	-0.5111	-1.179 to 0.1568	ns	0.2635
D1 T2 vs. D4 T2	0.8826	0.1503 to 1.615	**	0.0078
D2 T1 vs. D2 T2	1.939	0.9441 to 2.933	****	<0.0001
D2 T1 vs. D3 T1	-0.8432	-1.387 to -0.2993	***	0.0002
D2 T1 vs. D3 T2	1.349	0.3097 to 2.387	**	0.003
D2 T1 vs. D4 T1	0.6932	-0.3278 to 1.714	ns	0.4145
D2 T1 vs. D4 T2	2.122	0.9630 to 3.281	****	<0.0001
D2 T2 vs. D3 T1	-2.789	-3.906 to -1.673	****	<0.0001
D2 T2 vs. D3 T2	-0.6025	-1.012 to -0.1930	***	0.0004
D2 T2 vs. D4 T1	-1.253	-1.816 to -0.6889	****	<0.0001
D2 T2 vs. D4 T2	0.1253	-0.4501 to 0.7008	ns	0.9973
D3 T1 vs. D3 T2	2.164	1.070 to 3.258	****	<0.0001
D3 T1 vs. D4 T1	1.531	0.4028 to 2.660	**	0.0016
D3 T1 vs. D4 T2	3.024	1.761 to 4.287	****	<0.0001
D3 T2 vs. D4 T1	-0.65	-1.239 to -0.06126	*	0.0201
D3 T2 vs. D4 T2	0.7619	0.07520 to 1.449	*	0.0192
D4 T1 vs. D4 T2	1.434	0.7137 to 2.155	****	<0.0001

IL-18

Table S19. Descriptive statistics for daily cohort salivary levels of IL-18 at Time 1 (waking) and Time 2 (30 minutes post waking) during repeated acute stress.

Day	N, samples	Range (pg/mL)	Mean (pg/mL)	Std Deviation (pg/mL)	SEM (pg/mL)
Day 1	79	827.1	199.8	175.3	19.72
	76	808.1	111.9	132.5	15.2
Day 2	78	936.9	219.8	191.7	21.71
	79	340.6	69.57	58.49	6.581
Day 3	78	1202	274.4	214.9	24.33
	79	397.9	84.89	73.44	8.263
Day 4	79	831.1	213.6	159.5	17.94
	75	268.2	71.92	60.82	7.023

Table S20. Results of Tukey's multiple comparison tests between cohort average salivary IL-18 levels at each measurement point Days 1-4, with sampling at Time 1 (waking) and Time 2 (30 minutes post waking) each day during repeated acute stress.

Tukey's multiple comparisons test	Mean Diff.	95.00% CI of diff.	Summary	Adjusted P Value
D1 T1 vs. D1 T2	91.55	50.79 to 132.3	****	<0.0001
D1 T1 vs. D2 T1	-17.98	-60.70 to 24.74	ns	0.8919
D1 T1 vs. D2 T2	130.2	72.29 to 188.1	****	<0.0001
D1 T1 vs. D3 T1	-72.51	-126.4 to -18.60	**	0.0018
D1 T1 vs. D3 T2	114.9	53.08 to 176.7	****	<0.0001
D1 T1 vs. D4 T1	-13.86	-55.41 to 27.69	ns	0.9669
D1 T1 vs. D4 T2	118.8	60.28 to 177.3	****	<0.0001
D1 T2 vs. D2 T1	-110.1	-155.7 to -64.61	****	<0.0001
D1 T2 vs. D2 T2	42.07	3.494 to 80.64	*	0.0228
D1 T2 vs. D3 T1	-168.3	-222.3 to -114.3	****	<0.0001
D1 T2 vs. D3 T2	25.59	-22.75 to 73.92	ns	0.7183
D1 T2 vs. D4 T1	-104.2	-151.2 to -57.29	****	<0.0001
D1 T2 vs. D4 T2	35.51	-10.17 to 81.18	ns	0.2445
D2 T1 vs. D2 T2	150	89.77 to 210.2	****	<0.0001
D2 T1 vs. D3 T1	-53.42	-102.1 to -4.776	*	0.0213
D2 T1 vs. D3 T2	134.6	66.65 to 202.6	****	<0.0001
D2 T1 vs. D4 T1	4.39	-38.17 to 46.95	ns	>0.9999
D2 T1 vs. D4 T2	141.6	77.69 to 205.5	****	<0.0001
D2 T2 vs. D3 T1	-204.2	-271.8 to -136.5	****	<0.0001
D2 T2 vs. D3 T2	-15.32	-43.66 to 13.03	ns	0.6979
D2 T2 vs. D4 T1	-144.1	-196.9 to -91.23	****	<0.0001
D2 T2 vs. D4 T2	-1.477	-25.27 to 22.32	ns	>0.9999
D3 T1 vs. D3 T2	188.7	118.0 to 259.3	****	<0.0001
D3 T1 vs. D4 T1	58.31	7.946 to 108.7	*	0.0122
D3 T1 vs. D4 T2	199.1	123.2 to 275.1	****	<0.0001
D3 T2 vs. D4 T1	-128.8	-182.3 to -75.16	****	<0.0001
D3 T2 vs. D4 T2	15.64	-7.029 to 38.31	ns	0.3923
D4 T1 vs. D4 T2	140.9	85.48 to 196.2	****	<0.0001

MCP-1

Table S21. Descriptive statistics for daily cohort salivary levels of MCP-1 at Time 1 (waking) and Time 2 (30 minutes post waking) during repeated acute stress.

Day	N, samples	Range (pg/mL)	Mean (pg/mL)	Std Deviation (pg/mL)	SEM (pg/mL)
Day 1	78	3813	739.9	842.5	95.4
	76	2983	299.6	371.3	42.59
Day 2	78	4775	634.6	766.3	86.76
	79	1242	212	181.4	20.41
Day 3	77	2796	518.1	525.8	59.92
	79	1569	221.4	209.9	23.61
Day 4	79	3147	665.3	686.4	77.23
	75	631.2	199.1	137.7	15.9

Table S22. Results of Tukey's multiple comparison tests between cohort average salivary MCP-1 levels at each measurement point Days 1-4, with sampling at Time 1 (waking) and Time 2 (30 minutes post waking) each day during repeated acute stress.

Tukey's multiple comparisons test	Mean Diff.	95.00% CI of diff.	Summary	Adjusted P Value
D1 T1 vs. D1 T2	445.3	213.8 to 676.8	****	<0.0001
D1 T1 vs. D2 T1	163.5	3.331 to 323.6	*	0.042
D1 T1 vs. D2 T2	534.2	278.9 to 789.6	****	<0.0001
D1 T1 vs. D3 T1	227.2	23.04 to 431.3	*	0.0186
D1 T1 vs. D3 T2	535.9	272.0 to 799.8	****	<0.0001
D1 T1 vs. D4 T1	107.2	-83.79 to 298.3	ns	0.6554
D1 T1 vs. D4 T2	569.8	285.2 to 854.3	****	<0.0001
D1 T2 vs. D2 T1	-315.3	-493.0 to -137.5	****	<0.0001
D1 T2 vs. D2 T2	88.03	-15.02 to 191.1	ns	0.1503
D1 T2 vs. D3 T1	-246	-393.9 to -98.13	****	<0.0001
D1 T2 vs. D3 T2	78.35	7.223 to 149.5	*	0.0207
D1 T2 vs. D4 T1	-351	-527.6 to -174.5	****	<0.0001
D1 T2 vs. D4 T2	105.7	-4.754 to 216.1	ns	0.0707
D2 T1 vs. D2 T2	423.7	199.4 to 648.1	****	<0.0001
D2 T1 vs. D3 T1	68.33	-78.13 to 214.8	ns	0.8283
D2 T1 vs. D3 T2	413.2	202.6 to 623.9	****	<0.0001
D2 T1 vs. D4 T1	-31.14	-199.7 to 137.4	ns	0.9991
D2 T1 vs. D4 T2	450.3	196.9 to 703.8	****	<0.0001
D2 T2 vs. D3 T1	-311.7	-461.1 to -162.2	****	<0.0001
D2 T2 vs. D3 T2	-9.42	-58.09 to 39.25	ns	0.9987
D2 T2 vs. D4 T1	-453.3	-654.2 to -252.4	****	<0.0001
D2 T2 vs. D4 T2	17.2	-30.38 to 64.78	ns	0.9487
D3 T1 vs. D3 T2	313.4	166.3 to 460.4	****	<0.0001
D3 T1 vs. D4 T1	-116.3	-256.4 to 23.83	ns	0.177
D3 T1 vs. D4 T2	336.1	163.6 to 508.6	****	<0.0001
D3 T2 vs. D4 T1	-443.9	-636.3 to -251.5	****	<0.0001
D3 T2 vs. D4 T2	25.73	-26.98 to 78.44	ns	0.7928
D4 T1 vs. D4 T2	479.9	255.9 to 703.9	****	<0.0001

PDGF-AA

Table S23. Descriptive statistics for daily cohort salivary levels of PDGF-AA at Time 1 (waking) and Time 2 (30 minutes post waking) during repeated acute stress.

Day	N, samples	Range (pg/mL)	Mean (pg/mL)	Std Deviation (pg/mL)	SEM (pg/mL)
Day 1	76	3566	181.8	483.8	55.5
	76	1689	210.1	293.8	33.7
Day 2	75	1761	165.1	313.4	36.19
	79	1185	241.5	298.1	33.54
Day 3	77	1298	128	230.1	26.22
	79	1802	249.1	339.8	38.23
Day 4	78	493.3	83.52	108.1	12.24
	75	1058	176.6	241	27.83

Table S24. Results of Tukey's multiple comparison tests between cohort average salivary PDGF-AA levels at each measurement point Days 1-4, with sampling at Time 1 (waking) and Time 2 (30 minutes post waking) each day during repeated acute stress.

Tukey's multiple comparisons test	Mean Diff.	95.00% CI of diff.	Summary	Adjusted P Value
D1 T1 vs. D1 T2	-90.23	-192.6 to 12.12	ns	0.124
D1 T1 vs. D2 T1	18.59	-80.11 to 117.3	ns	0.9989
D1 T1 vs. D2 T2	-67.56	-229.2 to 94.07	ns	0.8948
D1 T1 vs. D3 T1	53.05	-102.2 to 208.3	ns	0.9618
D1 T1 vs. D3 T2	-76.24	-229.9 to 77.41	ns	0.7792
D1 T1 vs. D4 T1	97.34	-59.25 to 253.9	ns	0.5293
D1 T1 vs. D4 T2	1.392	-154.3 to 157.1	ns	>0.9999
D1 T2 vs. D2 T1	80.76	6.541 to 155.0	*	0.0234
D1 T2 vs. D2 T2	-12.01	-105.8 to 81.80	ns	>0.9999
D1 T2 vs. D3 T1	99.41	17.73 to 181.1	**	0.0069
D1 T2 vs. D3 T2	-31.1	-125.9 to 63.68	ns	0.9694
D1 T2 vs. D4 T1	133.1	41.69 to 224.5	***	0.0005
D1 T2 vs. D4 T2	43.4	-50.43 to 137.2	ns	0.8336
D2 T1 vs. D2 T2	-87.25	-195.5 to 21.02	ns	0.2061
D2 T1 vs. D3 T1	34.99	-41.01 to 111.0	ns	0.8371
D2 T1 vs. D3 T2	-93.47	-188.3 to 1.338	ns	0.0561
D2 T1 vs. D4 T1	79.99	-11.23 to 171.2	ns	0.1284
D2 T1 vs. D4 T2	-17.55	-111.9 to 76.76	ns	0.999
D2 T2 vs. D3 T1	119.5	29.24 to 209.7	**	0.0023
D2 T2 vs. D3 T2	-7.665	-100.4 to 85.04	ns	>0.9999
D2 T2 vs. D4 T1	160.8	74.53 to 247.1	****	<0.0001
D2 T2 vs. D4 T2	59.99	-9.610 to 129.6	ns	0.1425
D3 T1 vs. D3 T2	-127.3	-208.1 to -46.46	***	0.0001
D3 T1 vs. D4 T1	43.94	-20.16 to 108.0	ns	0.4011
D3 T1 vs. D4 T2	-51.49	-138.3 to 35.28	ns	0.5871
D3 T2 vs. D4 T1	168.6	67.60 to 269.6	****	<0.0001
D3 T2 vs. D4 T2	68.27	-15.22 to 151.8	ns	0.1912
D4 T1 vs. D4 T2	-94.9	-160.0 to -29.84	***	0.0005

TGFα

Table S25. Descriptive statistics for daily cohort salivary levels of TGFα at Time 1 (waking) and Time 2 (30 minutes post waking) during repeated acute stress.

Day	N, samples	Range (pg/mL)	Mean (pg/mL)	Std Deviation (pg/mL)	SEM (pg/mL)
Day 1	79	45.18	8.106	6.154	0.6924
	76	26.26	6.462	4.389	0.5035
Day 2	77	35.82	6.911	5.764	0.6569
	79	15.64	5.161	3.072	0.3456
Day 3	77	34.78	7.158	5.466	0.6229
	76	15.46	5.426	2.73	0.3131
Day 4	78	38.07	6.438	5.895	0.6675
	73	19.93	5.069	3.331	0.3899

Table S26. Results of Tukey's multiple comparison tests between cohort average salivary TGFα levels at each measurement point Days 1-4, with sampling at Time 1 (waking) and Time 2 (30 minutes post waking) each day during repeated acute stress.

Tukey's multiple comparisons test	Mean Diff.	95.00% CI of diff.	Summary	Adjusted P Value
D1 T1 vs. D1 T2	1.735	-0.1779 to 3.648	ns	0.1035
D1 T1 vs. D2 T1	1.309	0.01860 to 2.600	*	0.0443
D1 T1 vs. D2 T2	2.945	0.9104 to 4.980	***	0.0006
D1 T1 vs. D3 T1	1.04	-0.07695 to 2.157	ns	0.0864
D1 T1 vs. D3 T2	2.882	0.9990 to 4.765	***	0.0002
D1 T1 vs. D4 T1	1.753	0.2164 to 3.289	*	0.0143
D1 T1 vs. D4 T2	3.321	1.074 to 5.568	***	0.0004
D1 T2 vs. D2 T1	-0.4304	-2.318 to 1.457	ns	0.9964
D1 T2 vs. D2 T2	1.348	0.03127 to 2.665	*	0.041
D1 T2 vs. D3 T1	-0.7188	-2.490 to 1.052	ns	0.9081
D1 T2 vs. D3 T2	1.159	0.03656 to 2.282	*	0.0381
D1 T2 vs. D4 T1	0.008933	-2.197 to 2.215	ns	>0.9999
D1 T2 vs. D4 T2	1.512	-0.08654 to 3.110	ns	0.077
D2 T1 vs. D2 T2	1.671	-0.1226 to 3.464	ns	0.0861
D2 T1 vs. D3 T1	-0.2082	-0.9510 to 0.5347	ns	0.9875
D2 T1 vs. D3 T2	1.575	-0.09689 to 3.246	ns	0.0794
D2 T1 vs. D4 T1	0.4171	-1.129 to 1.963	ns	0.99
D2 T1 vs. D4 T2	1.973	-0.1187 to 4.065	ns	0.0785
D2 T2 vs. D3 T1	-1.895	-3.665 to -0.1253	*	0.0272
D2 T2 vs. D3 T2	-0.1187	-0.8786 to 0.6412	ns	0.9997
D2 T2 vs. D4 T1	-1.227	-3.094 to 0.6405	ns	0.4581
D2 T2 vs. D4 T2	0.1285	-0.9311 to 1.188	ns	>0.9999
D3 T1 vs. D3 T2	1.806	0.2596 to 3.352	*	0.0111
D3 T1 vs. D4 T1	0.6782	-0.8228 to 2.179	ns	0.8504
D3 T1 vs. D4 T2	2.245	0.3019 to 4.189	*	0.0126
D3 T2 vs. D4 T1	-1.09	-2.933 to 0.7529	ns	0.5925
D3 T2 vs. D4 T2	0.4344	-0.5890 to 1.458	ns	0.8858
D4 T1 vs. D4 T2	1.513	-0.5906 to 3.617	ns	0.338

VEGF-A

Table 27. Descriptive statistics for daily cohort salivary levels of VEGF-A at Time 1 (waking) and Time 2 (30 minutes post waking) during repeated acute stress.

Day	N, samples	Range (pg/mL)	Mean (pg/mL)	Std Deviation (pg/mL)	SEM (pg/mL)
Day 1	75	953	83.3	160.8	18.56
	69	718.7	130	168.4	20.27
Day 2	71	973.9	90.11	167.8	19.91
	72	737.6	171.7	203.4	23.97
Day 3	77	674.6	53.5	108.2	12.33
	76	869.7	176.7	200.4	22.98
Day 4	72	509.6	72.91	117.5	13.85
	70	693	132	172	20.56

Table S28. Results of Tukey's multiple comparison tests between cohort average salivary VEGF-A levels at each measurement point Days 1-4, with sampling at Time 1 (waking) and Time 2 (30 minutes post waking) each day during repeated acute stress.

Tukey's multiple comparisons test	Mean Diff.	95.00% CI of diff.	Summary	Adjusted P Value
D1 T1 vs. D1 T2	-61.15	-110.6 to -11.66	**	0.0059
D1 T1 vs. D2 T1	-2.355	-33.11 to 28.40	ns	>0.9999
D1 T1 vs. D2 T2	-87.57	-159.7 to -15.46	**	0.0072
D1 T1 vs. D3 T1	29.28	-19.49 to 78.04	ns	0.5727
D1 T1 vs. D3 T2	-99.15	-174.9 to -23.41	**	0.0027
D1 T1 vs. D4 T1	14.12	-42.12 to 70.36	ns	0.9934
D1 T1 vs. D4 T2	-47.52	-120.0 to 24.99	ns	0.456
D1 T2 vs. D2 T1	54.98	-0.7049 to 110.7	ns	0.0554
D1 T2 vs. D2 T2	-38.62	-115.5 to 38.23	ns	0.7628
D1 T2 vs. D3 T1	81.4	25.11 to 137.7	***	0.0006
D1 T2 vs. D3 T2	-55.17	-133.8 to 23.45	ns	0.3676
D1 T2 vs. D4 T1	59.32	4.479 to 114.2	*	0.0251
D1 T2 vs. D4 T2	0.1788	-63.18 to 63.54	ns	>0.9999
D2 T1 vs. D2 T2	-85.08	-157.4 to -12.72	*	0.0105
D2 T1 vs. D3 T1	32.86	-10.18 to 75.90	ns	0.2645
D2 T1 vs. D3 T2	-97.82	-172.8 to -22.88	**	0.0029
D2 T1 vs. D4 T1	17.87	-41.06 to 76.81	ns	0.9796
D2 T1 vs. D4 T2	-49.65	-122.4 to 23.10	ns	0.4014
D2 T2 vs. D3 T1	118.1	48.46 to 187.8	****	<0.0001
D2 T2 vs. D3 T2	-12.34	-74.64 to 49.97	ns	0.9985
D2 T2 vs. D4 T1	107.3	41.62 to 173.1	****	<0.0001
D2 T2 vs. D4 T2	34.35	-20.64 to 89.34	ns	0.5181
D3 T1 vs. D3 T2	-125.5	-190.9 to -60.15	****	<0.0001
D3 T1 vs. D4 T1	-16.82	-52.42 to 18.77	ns	0.8165
D3 T1 vs. D4 T2	-79.47	-140.5 to -18.42	**	0.003
D3 T2 vs. D4 T1	114.1	50.33 to 177.9	****	<0.0001
D3 T2 vs. D4 T2	45.04	-9.385 to 99.46	ns	0.1784
D4 T1 vs. D4 T2	-65.46	-123.2 to -7.759	*	0.0155

TNFα

Table S29. Descriptive statistics for daily cohort salivary levels of TNFα at Time 1 (waking) and Time 2 (30 minutes post waking) during repeated acute stress.

Day	N, samples	Range (pg/mL)	Mean (pg/mL)	Std Deviation (pg/mL)	SEM (pg/mL)
Day 1	36	34.51	8.575	7.952	1.325
	34	6.93	3.601	1.738	0.2981
Day 2	38	30.31	6.891	7.173	1.164
	31	12.72	3.455	2.505	0.4499
Day 3	38	21.96	7.346	4.886	0.7926
	37	9.89	3.316	1.917	0.3152
Day 4	40	28.66	5.436	5.151	0.8144
	30	9.97	3.101	2.022	0.3692

Table S30. Results of Tukey's multiple comparison tests between cohort average salivary TNFα levels at each measurement point Days 1-4, with sampling at Time 1 (waking) and Time 2 (30 minutes post waking) each day during repeated acute stress.

Tukey's multiple comparisons test	Mean Diff.	95.00% CI of diff.	Summary	Adjusted P Value
D1 T1 vs. D1 T2	5.775	1.599 to 9.951	**	0.0021
D1 T1 vs. D2 T1	1.253	-2.382 to 4.889	ns	0.9496
D1 T1 vs. D2 T2	6.097	1.289 to 10.91	**	0.006
D1 T1 vs. D3 T1	0.9353	-2.431 to 4.302	ns	0.9842
D1 T1 vs. D3 T2	5.481	1.253 to 9.708	**	0.0043
D1 T1 vs. D4 T1	2.794	-1.364 to 6.951	ns	0.3965
D1 T1 vs. D4 T2	6.901	1.728 to 12.08	**	0.0035
D1 T2 vs. D2 T1	-4.135	-7.770 to -0.5002	*	0.017
D1 T2 vs. D2 T2	0.2369	-0.9387 to 1.412	ns	0.9975
D1 T2 vs. D3 T1	-4.132	-6.581 to -1.682	***	0.0001
D1 T2 vs. D3 T2	0.1803	-0.7052 to 1.066	ns	0.9975
D1 T2 vs. D4 T1	-2.396	-4.948 to 0.1552	ns	0.0783
D1 T2 vs. D4 T2	0.7948	-0.3289 to 1.919	ns	0.3218
D2 T1 vs. D2 T2	4.546	0.9880 to 8.104	**	0.0055
D2 T1 vs. D3 T1	-0.3883	-3.489 to 2.712	ns	>0.9999
D2 T1 vs. D3 T2	3.922	0.4200 to 7.423	*	0.0193
D2 T1 vs. D4 T1	1.452	-0.8278 to 3.731	ns	0.4677
D2 T1 vs. D4 T2	5.288	1.248 to 9.327	**	0.0044
D2 T2 vs. D3 T1	-4.595	-7.452 to -1.737	***	0.0003
D2 T2 vs. D3 T2	-0.1777	-1.187 to 0.8314	ns	0.9989
D2 T2 vs. D4 T1	-2.749	-5.081 to -0.4164	*	0.0123
D2 T2 vs. D4 T2	0.4474	-0.3793 to 1.274	ns	0.6379
D3 T1 vs. D3 T2	4.236	1.813 to 6.659	****	<0.0001
D3 T1 vs. D4 T1	1.723	-1.067 to 4.513	ns	0.5063
D3 T1 vs. D4 T2	5.259	2.399 to 8.119	****	<0.0001
D3 T2 vs. D4 T1	-2.399	-4.684 to -0.1140	*	0.0339
D3 T2 vs. D4 T2	0.5377	-0.2735 to 1.349	ns	0.4022
D4 T1 vs. D4 T2	3.326	0.8749 to 5.776	**	0.0028

CX3CL1

Table S31. Descriptive statistics for daily cohort salivary levels of CX3CL1 at Time 1 (waking) and Time 2 (30 minutes post waking) during repeated acute stress.

Day	N, samples	Range (pg/mL)	Mean (pg/mL)	Std Deviation (pg/mL)	SEM (pg/mL)
Day 1	39	46.36	11.63	9.255	1.482
	40	26.61	7.829	7.156	1.131
Day 2	38	26.61	9.007	7.271	1.18
	38	21.53	4.987	5.414	0.8782
Day 3	38	44.33	13.54	8.969	1.455
	37	44.33	8.424	8.366	1.375
Day 4	39	31.36	7.685	8.927	1.429
	40	18.82	4.905	5.933	0.9381

Table S32. Results of Tukey's multiple comparison tests between cohort average salivary CX3CL1 levels at each measurement point Days 1-4, with sampling at Time 1 (waking) and Time 2 (30 minutes post waking) each day during repeated acute stress.

Tukey's multiple comparisons test	Mean Diff.	95.00% CI of diff.	Summary	Adjusted P Value
D1 T1 vs. D1 T2	3.6	-0.2711 to 7.471	ns	0.0844
D1 T1 vs. D2 T1	3.007	-1.096 to 7.111	ns	0.2928
D1 T1 vs. D2 T2	6.878	1.936 to 11.82	**	0.0017
D1 T1 vs. D3 T1	-1.758	-7.374 to 3.858	ns	0.9705
D1 T1 vs. D3 T2	2.897	-3.005 to 8.800	ns	0.758
D1 T1 vs. D4 T1	4.047	-1.750 to 9.845	ns	0.352
D1 T1 vs. D4 T2	6.598	2.022 to 11.17	**	0.001
D1 T2 vs. D2 T1	-0.9692	-4.435 to 2.497	ns	0.9845
D1 T2 vs. D2 T2	3.002	-0.05686 to 6.061	ns	0.0576
D1 T2 vs. D3 T1	-5.454	-9.787 to -1.121	**	0.0057
D1 T2 vs. D3 T2	-0.7278	-4.742 to 3.286	ns	0.9989
D1 T2 vs. D4 T1	0.1977	-3.970 to 4.365	ns	>0.9999
D1 T2 vs. D4 T2	2.924	-0.9419 to 6.789	ns	0.2601
D2 T1 vs. D2 T2	3.716	0.3865 to 7.046	*	0.0198
D2 T1 vs. D3 T1	-4.366	-7.954 to -0.7779	**	0.0084
D2 T1 vs. D3 T2	0.2466	-4.750 to 5.244	ns	>0.9999
D2 T1 vs. D4 T1	1.149	-2.185 to 4.484	ns	0.9508
D2 T1 vs. D4 T2	3.844	0.3039 to 7.384	*	0.0253
D2 T2 vs. D3 T1	-8.352	-12.59 to -4.116	****	<0.0001
D2 T2 vs. D3 T2	-3.875	-7.818 to 0.06926	ns	0.0571
D2 T2 vs. D4 T1	-2.476	-6.727 to 1.774	ns	0.5769
D2 T2 vs. D4 T2	0.07553	-3.061 to 3.212	ns	>0.9999
D3 T1 vs. D3 T2	4.835	-0.5531 to 10.22	ns	0.1053
D3 T1 vs. D4 T1	5.461	1.278 to 9.645	**	0.0038
D3 T1 vs. D4 T2	8.382	3.898 to 12.87	****	<0.0001
D3 T2 vs. D4 T1	0.505	-5.491 to 6.501	ns	>0.9999
D3 T2 vs. D4 T2	3.33	-0.8669 to 7.527	ns	0.2074
D4 T1 vs. D4 T2	2.655	-2.117 to 7.427	ns	0.6346

G-CSF

Table S33. Descriptive statistics for daily cohort salivary levels of G-CSF at Time 1 (waking) and Time 2 (30 minutes post waking) during repeated acute stress.

Day	N, samples	Range (pg/mL)	Mean (pg/mL)	Std Deviation (pg/mL)	SEM (pg/mL)
Day 1	39	223	52.5	47.81	7.656
	39	332.3	54.55	63.4	10.15
Day 2	39	99.76	33.95	21.61	3.46
	39	181.7	36.07	35.12	5.624
Day 3	39	165	44.36	27.83	4.457
	40	156.1	48.93	36.2	5.724
Day 4	40	90.9	32.15	19.5	3.084
	40	125.4	31.99	27.62	4.366

Table S34. Results of Tukey's multiple comparison tests between cohort average salivary G-CSF levels at each measurement point Days 1-4, with sampling at Time 1 (waking) and Time 2 (30 minutes post waking) each day during repeated acute stress.

Tukey's multiple comparisons test	Mean Diff.	95.00% CI of diff.	Summary	Adjusted P Value
D1 T1 vs. D1 T2	-2.804	-38.87 to 33.27	ns	>0.9999
D1 T1 vs. D2 T1	18.35	-3.576 to 40.28	ns	0.1588
D1 T1 vs. D2 T2	16.43	-7.650 to 40.50	ns	0.3818
D1 T1 vs. D3 T1	8.068	-10.33 to 26.47	ns	0.8479
D1 T1 vs. D3 T2	2.316	-21.05 to 25.68	ns	>0.9999
D1 T1 vs. D4 T1	19.68	-1.746 to 41.11	ns	0.0916
D1 T1 vs. D4 T2	19.72	-3.304 to 42.73	ns	0.1403
D1 T2 vs. D2 T1	21.24	-9.815 to 52.29	ns	0.3775
D1 T2 vs. D2 T2	19.39	-15.25 to 54.03	ns	0.626
D1 T2 vs. D3 T1	9.858	-21.57 to 41.28	ns	0.9704
D1 T2 vs. D3 T2	4.667	-17.64 to 26.98	ns	0.9973
D1 T2 vs. D4 T1	22.05	-9.104 to 53.21	ns	0.3364
D1 T2 vs. D4 T2	22.08	-2.960 to 47.12	ns	0.1183
D2 T1 vs. D2 T2	-1.362	-13.87 to 11.15	ns	>0.9999
D2 T1 vs. D3 T1	-10.56	-19.58 to -1.533	*	0.0125
D2 T1 vs. D3 T2	-14.26	-28.60 to 0.08141	ns	0.0522
D2 T1 vs. D4 T1	1.818	-5.761 to 9.398	ns	0.9938
D2 T1 vs. D4 T2	2.001	-8.535 to 12.54	ns	0.9985
D2 T2 vs. D3 T1	-9.192	-25.45 to 7.065	ns	0.6142
D2 T2 vs. D3 T2	-14.11	-31.34 to 3.123	ns	0.1788
D2 T2 vs. D4 T1	3.258	-10.56 to 17.07	ns	0.9944
D2 T2 vs. D4 T2	3.289	-12.37 to 18.95	ns	0.9973
D3 T1 vs. D3 T2	-5.342	-19.99 to 9.306	ns	0.9358
D3 T1 vs. D4 T1	11.78	2.959 to 20.60	**	0.0028
D3 T1 vs. D4 T2	11.99	0.5796 to 23.41	*	0.0335
D3 T2 vs. D4 T1	16.78	2.561 to 31.00	*	0.0113
D3 T2 vs. D4 T2	16.94	6.601 to 27.28	***	0.0001
D4 T1 vs. D4 T2	0.16	-9.849 to 10.17	ns	>0.9999

INF-γ

Table S35. Descriptive statistics for daily cohort salivary levels of INFγ at Time 1 (waking) and Time 2 (30 minutes post waking) during repeated acute stress.

Day	N, samples	Range (pg/mL)	Mean (pg/mL)	Std Deviation (pg/mL)	SEM (pg/mL)
Day 1	40	125.4	31.99	27.62	4.366
	38	2.06	0.8374	0.4724	0.07664
Day 2	38	22.12	1.757	3.53	0.5727
	30	3.58	0.71	0.6665	0.1217
Day 3	39	4.19	1.569	0.8676	0.1389
	36	2.12	0.8317	0.5131	0.08552
Day 4	39	3.03	1.164	0.6775	0.1085
	29	1.6	0.6503	0.4235	0.07864

Table S36. Results of Tukey's multiple comparison tests between cohort average salivary IFNγ levels at each measurement point Days 1-4, with sampling at Time 1 (waking) and Time 2 (30 minutes post waking) each day during repeated acute stress.

Tukey's multiple comparisons test	Mean Diff.	95.00% CI of diff.	Summary	Adjusted P Value
D1 T1 vs. D1 T2	30.64	16.25 to 45.03	****	<0.0001
D1 T1 vs. D2 T1	30.91	16.96 to 44.86	****	<0.0001
D1 T1 vs. D2 T2	37.09	20.03 to 54.15	****	<0.0001
D1 T1 vs. D3 T1	30.8	16.63 to 44.97	****	<0.0001
D1 T1 vs. D3 T2	32.92	17.65 to 48.18	****	<0.0001
D1 T1 vs. D4 T1	31.21	16.99 to 45.44	****	<0.0001
D1 T1 vs. D4 T2	31.82	16.84 to 46.80	****	<0.0001
D1 T2 vs. D2 T1	-0.9367	-2.789 to 0.9155	ns	0.7302
D1 T2 vs. D2 T2	0.2386	-0.08383 to 0.5610	ns	0.2691
D1 T2 vs. D3 T1	-0.7397	-1.180 to -0.2991	***	0.0001
D1 T2 vs. D3 T2	0.02657	-0.2663 to 0.3195	ns	>0.9999
D1 T2 vs. D4 T1	-0.3297	-0.6912 to 0.03173	ns	0.0954
D1 T2 vs. D4 T2	0.2357	-0.04284 to 0.5143	ns	0.1436
D2 T1 vs. D2 T2	1.379	-0.7067 to 3.465	ns	0.4029
D2 T1 vs. D3 T1	0.19	-1.666 to 2.046	ns	>0.9999
D2 T1 vs. D3 T2	1.037	-1.001 to 3.075	ns	0.7205
D2 T1 vs. D4 T1	0.6222	-1.182 to 2.426	ns	0.9506
D2 T1 vs. D4 T2	1.449	-1.070 to 3.967	ns	0.5679
D2 T2 vs. D3 T1	-1.075	-1.687 to -0.4623	****	<0.0001
D2 T2 vs. D3 T2	-0.2046	-0.6538 to 0.2445	ns	0.8038
D2 T2 vs. D4 T1	-0.6057	-1.097 to -0.1138	**	0.008
D2 T2 vs. D4 T2	0.06875	-0.3380 to 0.4755	ns	0.999
D3 T1 vs. D3 T2	0.7994	0.4827 to 1.116	****	<0.0001
D3 T1 vs. D4 T1	0.4176	0.02456 to 0.8107	*	0.0306
D3 T1 vs. D4 T2	0.9755	0.6313 to 1.320	****	<0.0001
D3 T2 vs. D4 T1	-0.3597	0.7251 to 0.005635	ns	0.0562
D3 T2 vs. D4 T2	0.1961	-0.04166 to 0.4338	ns	0.1643
D4 T1 vs. D4 T2	0.6264	0.3280 to 0.9249	****	<0.0001

IL-1β

Table S37. Descriptive statistics for daily cohort salivary levels of IL-1β at Time 1 (waking) and Time 2 (30 minutes post waking) during repeated acute stress.

Day	N, samples	Range (pg/mL)	Mean (pg/mL)	Std Deviation (pg/mL)	SEM (pg/mL)
Day 1	40	1429	306.3	328.1	51.87
	40	341.7	66.09	88.1	13.93
Day 2	39	2611	418.6	460.9	73.8
	40	593.2	83.48	115	18.18
Day 3	39	2500	453.9	443.7	71.04
	39	761.3	78.8	124.1	19.87
Day 4	40	2553	368.5	430	67.99
	40	745.8	79.48	129.3	20.44

Table S38. Results of Tukey's multiple comparison tests between cohort average salivary IL-1β levels at each measurement point Days 1-4, with sampling at Time 1 (waking) and Time 2 (30 minutes post waking) each day during repeated acute stress.

Tukey's multiple comparisons test	Mean Diff.	95.00% CI of diff.	Summary	Adjusted P Value
D1 T1 vs. D1 T2	240.2	96.29 to 384.1	***	0.0001
D1 T1 vs. D2 T1	-106.3	-304.8 to 92.14	ns	0.6764
D1 T1 vs. D2 T2	222.8	80.55 to 365.0	***	0.0003
D1 T1 vs. D3 T1	-140.6	-336.3 to 55.13	ns	0.3187
D1 T1 vs. D3 T2	234.8	73.70 to 395.8	***	0.0009
D1 T1 vs. D4 T1	-62.2	-280.4 to 156.0	ns	0.983
D1 T1 vs. D4 T2	226.8	63.11 to 390.5	**	0.0017
D1 T2 vs. D2 T1	-351	-558.1 to -143.9	****	<0.0001
D1 T2 vs. D2 T2	-17.39	-43.48 to 8.705	ns	0.4129
D1 T2 vs. D3 T1	-386.4	-591.0 to -181.7	****	<0.0001
D1 T2 vs. D3 T2	-11.08	-60.62 to 38.46	ns	0.996
D1 T2 vs. D4 T1	-302.4	-499.2 to -105.6	***	0.0004
D1 T2 vs. D4 T2	-13.38	-63.41 to 36.64	ns	0.9882
D2 T1 vs. D2 T2	333.2	143.4 to 522.9	****	<0.0001
D2 T1 vs. D3 T1	-37.44	-139.3 to 64.40	ns	0.9326
D2 T1 vs. D3 T2	348.2	162.0 to 534.4	****	<0.0001
D2 T1 vs. D4 T1	42.71	-75.86 to 161.3	ns	0.9397
D2 T1 vs. D4 T2	337.7	152.7 to 522.7	****	<0.0001
D2 T2 vs. D3 T1	-368.5	-553.7 to -183.4	****	<0.0001
D2 T2 vs. D3 T2	6.768	-30.01 to 43.55	ns	0.9988
D2 T2 vs. D4 T1	-285	-464.9 to -105.1	***	0.0002
D2 T2 vs. D4 T2	4.006	-36.55 to 44.56	ns	>0.9999
D3 T1 vs. D3 T2	382.4	204.7 to 560.1	****	<0.0001
D3 T1 vs. D4 T1	78.77	-41.88 to 199.4	ns	0.4375
D3 T1 vs. D4 T2	372.8	189.4 to 556.2	****	<0.0001
D3 T2 vs. D4 T1	-293.4	-461.3 to -125.4	****	<0.0001
D3 T2 vs. D4 T2	-2.375	-34.49 to 29.74	ns	>0.9999
D4 T1 vs. D4 T2	289	118.2 to 459.8	****	<0.0001
